# Design, Fabrication, and Application of Stretchable Electronic Conductors

**DOI:** 10.1007/s40820-025-02009-3

**Published:** 2026-01-05

**Authors:** Bin Cheng, Jingting Zhuo, Yao Zhou, Jiaxiang Chen, Lingyun Cao, Jiangfeng He, Zhihong Chen, Xiaoxiao Ma, Juan Wang, Honglong Li, Guowei Yang, Fang Yi

**Affiliations:** School of Materials Science and Engineering, Nanotechnology Research Center, State Key Laboratory of Optoelectronic Materials and Technologies, Guangdong Engineering Technology Research Center for Functional Biomaterials, Guangzhou Key Laboratory of Flexible Electronic Materials and Wearable Devices, Sun Yat-Sen University, Guangzhou, 510275 People’s Republic of China

**Keywords:** Stretchable conductors, Electronic conductors, Stretchable electronics, Wearable electronics

## Abstract

Stretchable electronics have been recognized as intriguing next-generation electronics that possess huge market value, and stretchable electronic conductors (SECs) are essential for stretchable electronics, which not only can serve as critical functional components but also are the indispensable electronic connections bridging various electronic components within stretchable electronic systems. Herein, we offer a comprehensive review of recent progress in SECs including the material categories, structure designs, fabrication techniques, and applications. The characteristics, performance enhancement strategies, and application requirements are emphasized. Based on the recent advances, the existing challenges and future prospects are outlined and discussed.

## Highlights

- A comprehensive review of recent advances in stretchable electronic conductors including the material categories, structure designs, fabrication techniques, and applications.
- A novel emphasis on the characteristics, performance enhancement strategies, and application requirements of stretchable electronic conductors.
- An exhaustive analysis of the existing challenges and future prospects for stretchable electronic conductors.

## Graphical abstract

**Figure:**
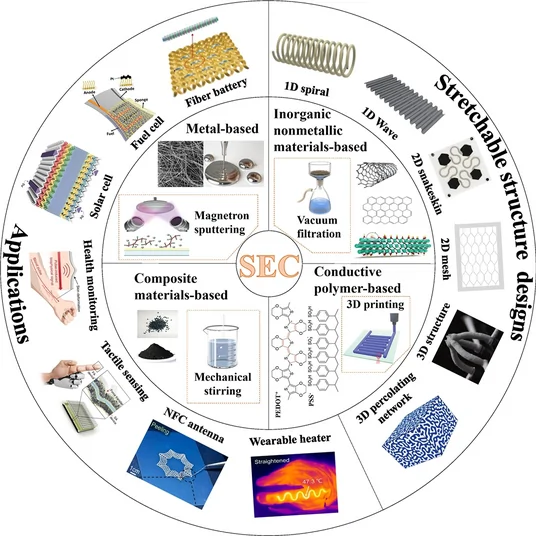

## Introduction

Traditional rigid electronics are uncapable of conforming to curved or deformable surfaces which are commonly seen in daily life, and stretchable electronics emerge to address such challenges, whose stretchability and shape adaption are mainly realized through stretchable structural designs and intrinsically stretchable materials. Stretchable electronics have been given substantial attention and shown tremendous potential to revolutionize myriad areas such as medical care, robotics, and sports [1–8]. As conductive materials that can maintain reliable electrical properties despite substantial mechanical deformation, stretchable conductors are critical base materials for stretchable electronics, which allow for seamless integration with various irregular surfaces and excellent adaptability to operational environments [9–12]. The conductive mechanisms in stretchable conductors can be divided into two categories: ionic conduction [13–15] and electronic conduction, with electronic conduction being more prevalent. Electronic conductivity is not only fundamental for the functionality of electronics but also directly influences their working performance, stability, and potential applications. Therefore, the research and development of stretchable electronic conductors (SECs) is of paramount importance [16–20]. SECs guarantee the realization of both basic functions and the integration of multiple functionalities within stretchable electronic systems. Beyond serving as the electrodes and conductive interconnecting components, they can offer sensitive sensing properties, adjustable thermal management, and effective electromagnetic interference shielding [21–27]. Particularly, the intrinsic electrical stability of SECs is crucial for ensuring normal operation and extending the service life of stretchable electronic devices. Transparent SECs offer visibility, which are in critical demand in applications such as displays and wearables [28–34]. The significance of SECs as one core material of stretchable electronics is underscored by the substantial technological innovation and increasing market value observed in recent years, and the breakthroughs in various fields, such as biomedicine, human–computer interaction, and energy management, not only deepen our basic understanding of SECs but also inject new momentum into the industrialization of stretchable electronic technology. SECs have been undergoing burgeoning development and inevitably facing challenges in practical applications. Hence, a comprehensive review of the research on SECs is both timely and critical [35–42].

**Fig. 1:**
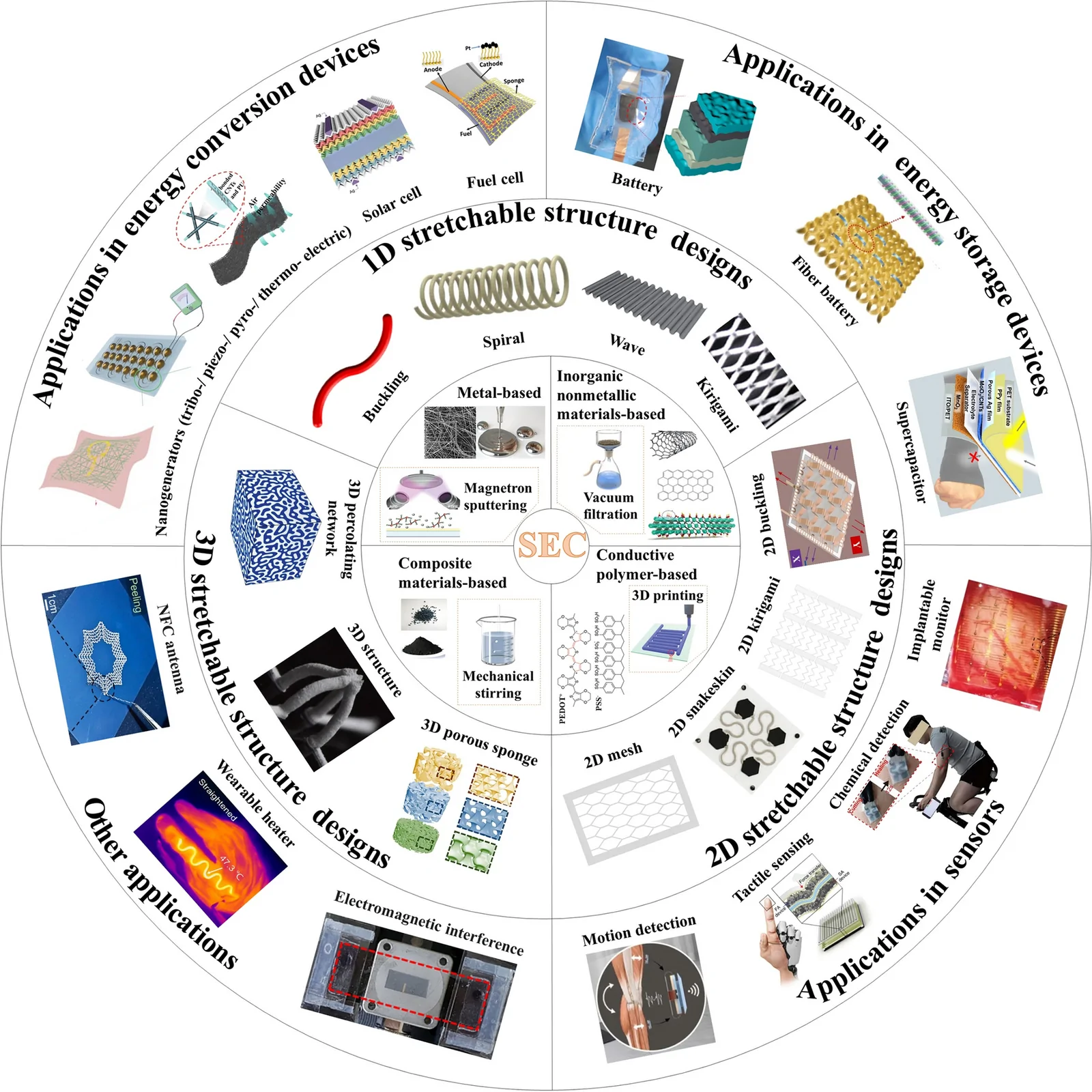
Overview of the categories, structure designs, fabrication techniques, and applications of SECs. Metal-based SECs. AgNWs: Reproduced with permission [177]. Copyright 2023, Wiley‐VCH GmbH. Magnetron sputtering: Reproduced with permission [26]. Copyright 2020, Wiley‐VCH GmbH. Inorganic nonmetallic materials-based SECs. CNT and graphene: Reproduced with permission [19]. Copyright 2021, MDPI, Basel, Switzerland. MXene: Reproduced with permission [20]. Copyright 2023, Wiley‐VCH GmbH. Vacuum filtration: Reproduced with permission [237]. Copyright 2024, Elsevier Ltd. Conductive polymer-based SECs. PEDOT:PSS: Reproduced with permission [252]. Copyright 2019, WILEY‐VCH Verlag GmbH & Co. KGaA, Weinheim. 3D Printing: Reproduced with permission [288]. Copyright 2020, WILEY‐VCH Verlag GmbH & Co. KGaA, Weinheim. 1D stretchable structure designs. Spiral: Reproduced with permission [42]. Copyright 2020, WILEY‐VCH Verlag GmbH & Co. KGaA, Weinheim. Wave: Reproduced with permission [194]. Copyright 2014, Royal Society of Chemistry. Kirigami: Reproduced with permission [199]. Copyright 2023, Wiley‐VCH GmbH. 2D stretchable structure designs. 2D buckling: Reproduced with permission [205]. Copyright 2022, Elsevier Ltd. 2D snakeskin: Reproduced with permission [209]. Copyright 2020, WILEY‐VCH Verlag GmbH & Co. KGaA, Weinheim. 3D stretchable structure designs. 3D porous sponge: Reproduced with permission [40]. Copyright 2024, Elsevier B.V. 3D structure: Reproduced with permission [218]. Copyright 2023, Springer Nature Limited. 3D percolating network: Reproduced with permission [41]. Copyright 2023, Springer Nature Limited. Applications in energy conversion devices. Nanogenerators (tribo-/piezo-/pyro-/thermo- electric): Reproduced with permission [310] [329]. Copyright 2022, Wiley‐VCH GmbH. Reproduced with permission [316]. Copyright 2024, Wiley‐VCH GmbH. Solar cell: Reproduced with permission [27]. Copyright 2024, Springer Nature Limited. Fuel cell: Reproduced with permission [356]. Copyright 2019, WILEY‐VCH Verlag GmbH & Co. KGaA, Weinheim. Applications in energy storage devices. Battery: Reproduced with permission [367]. Copyright 2024, American Chemical Society. Fiber battery: Reproduced with permission [378]. Copyright 2023, Donghua University, Shanghai, China. Supercapacitor: Reproduced with permission [388]. Copyright 2022, Elsevier B.V. Applications in sensors. Motion detection: Reproduced with permission [407]. Copyright 2025, Elsevier Ltd. Tactile sensing: Reproduced with permission [419]. Copyright 2021, Springer Nature Limited. Chemical detection: Reproduced with permission [422]. Copyright 2022, Elsevier B.V. Implantable monitor: Reproduced with permission [425]. Copyright 2023, Korean Society of Medical and Biological Engineering. Other applications. NFC antenna: Reproduced with permission [440]. Copyright 2023, Elsevier Ltd. Wearable heater: Reproduced with permission [439]. Copyright 2023, Elsevier B.V. Electromagnetic interference shielding: Reproduced with permission [29]. Copyright 2017, American Chemical Society

In this review, we will summarize the latest advances in the field of SECs, with an emphasis on their characteristics, performance enhancement strategies, and application requirements. As shown in the overview in Fig. 1, we will begin by providing an exhaustive review of commonly utilized types of materials for SECs, summarize the characteristics and advantages/disadvantages of different types of materials, and discuss plausible strategies for performance enhancement. Subsequently, we will overview and discuss the effect of structural design on the properties of SECs and analyze the reasons why specific structures lead to high performance. Following this, we will review the fabrication techniques employed for different types of SECs and summarize their pros and cons. Then, we will give a detailed review of the functions and requirements of SECs applied in diverse fields. Finally, we will outline each category of SECs, discuss the existing challenges, and offer a perspective on the future development prospects and application potentials of SECs.

## Classification of SECs

There have been a couple of classifications for SECs, such as those based on different structures and matrix materials. In this review, we categorize SECs based on their primary conductive components: metal-based, inorganic nonmetallic materials-based, conductive polymer-based, and composite materials-based SECs.

### Metal-Based SECs

Metal-based SECs can be divided into two types: solid metal-based and liquid metal (LM) -based SECs.

#### Solid Metal-Based SECs

Solid metal materials commonly refer to those metal materials that maintain a solid crystalline structure at room temperature and have high density and mechanical strength. To be applied in SECs, these materials are generally first processed into various nanostructures, such as ultra-thin metal films, metal nanofibers, metal nanogrooves, metal nanowires (NWs), metal nanoflakes, and metal nets, and then manufactured into designed shapes and combined with stretchable substrates [43–47]. The most commonly applied solid metal materials are noble metal nanomaterials like gold (Au) and silver (Ag) due to their inherent stability. Other metal nanomaterials, such as copper (Cu) and ferrum (Fe), demonstrate higher chemical activity, rendering them more susceptible to oxidation and instability. High aspect ratios are critical for metal NWs to achieve both high electrical conductivity and mechanical compliance [48–50]. It is worth noting that while noble metal nanomaterials generally exhibit good stability, they can still undergo oxidation or other chemical reactions under specific environments or conditions, including exposure to strong oxidants, high temperatures, and high humidity [51–53].

The conductivity of solid metal-based SECs under tensile deformation can be improved through various structural designs of solid metal nanomaterials.

**Fig. 2:**
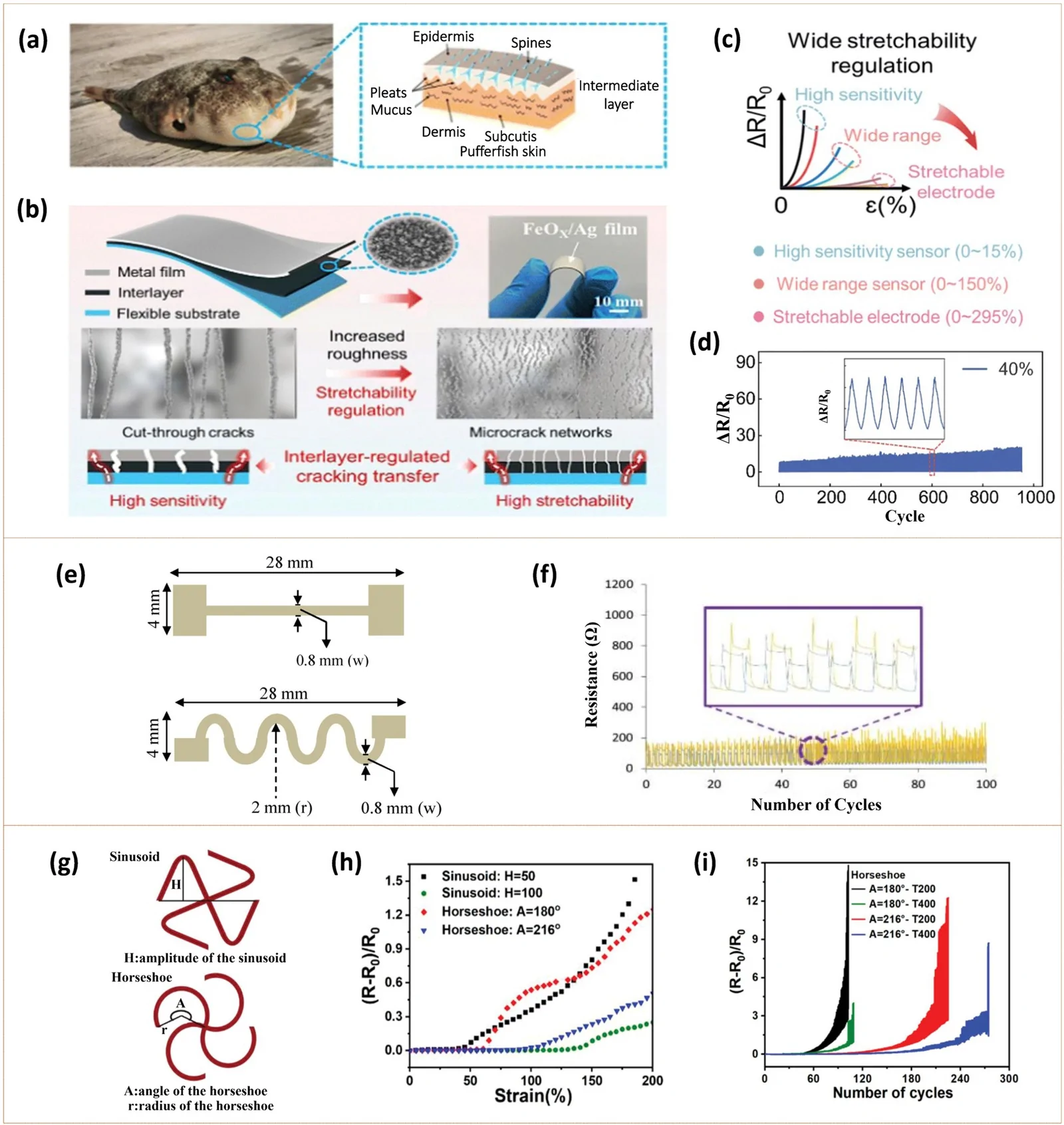
Solid metal-based SECs. **a** Photograph of a pufferfish and schematic diagram of the skin structure on its abdomen. **b** The pufferfish-inspired stretchable, stretchability-tunable metal films. The upper subfigures show the structure and picture of the fabricated metal films. The lower subfigures show the evolution of their crack pattern with an electromechanical performance transition from high sensitivity to high stretchability by the interlayer regulation strategy. **c** Three kinds of SECs based on metal films characterized by ΔR/R_0_ vs. strain (the high-sensitivity sensor, wide-range sensor, and stretchable electrode). **d** The repeated cycling test of the Ag/FeO_x_ film at a strain of 40% (loading speed: 0.5 mm s^−1^). Reproduced with permission [57]. Copyright 2023, Royal Society of Chemistry. **e** Schematic diagram of a straight-line and wavy-line configuration for the Ag/AgNWs SEC. **f** Electromechanical response of the SEC with a straight-line configuration during a cyclic stretching/releasing test (elongation: 3 mm; frequency: 3 Hz; 100 cycles). Reproduced with permission [61]. Copyright 2018, Elsevier B.V. **g** The principle and parameters of horseshoe-like and sinusoid-like metal mesh transparent SECs. **h** Variation in resistance vs. applied strain of the SECs based on different metal mesh structures. **i** SECs based on metal mesh structures with different deviation angles (0°, 15°, 30°, and 45°): electrical response under stretching/releasing cycles at 30% strain. Reproduced with permission [65]. Copyright 2023, Wiley‐VCH GmbH

1. Deposition of a micro-crack network pattern on a thin metal layer [54–57]. For example, inspired by the puffer fish, Sun et al. [57] proposed an interlayer adjustment strategy by introducing an intermediate layer (FeO_x_) between the polymer substrate and metal film to achieve stretchability (Fig. 2a–d). The Ag/FeO_x_ film was deposited on a polydimethylsiloxane (PDMS) substrate via a two-step deposition process. The surface roughness of FeO_x_ can be controlled by adjusting the deposition pressure. The strong interfacial adhesion between Ag and FeO_x_ layers facilitated the effective transfer of the crack mode of FeO_x_ to the metal film, enabling crack modulation. This approach resulted in a nearly 20-fold increase in the stretchability of the Ag film (maximum strain: 295%), while maintaining conductivity over 900 cycles at 40% strain.
2. Construction of hybrid structures combining metal nanomaterials with thin metal layers [58–61]. For example, Cho et al. [60] developed a type of SEC using metal/Ag NWs/metal hybrid structures on a PDMS substrate. Hybrid structures of Ag/AgNWs/Ag (ANA) and Cu/AgNWs/Cu electrodes achieved low sheet resistances of around 100 mΩ sq^−1^. The AgNWs between the top and bottom metal electrodes improved the tensile properties under both single and multi-cycling strain conditions. The randomly interconnected AgNWs generated a new conductive path across cracks and wavy structures in the metal electrodes, thereby enhancing the conductivity of these SECs under strain. Ali et al. [61] prepared an SEC by screen printing Ag/AgNWs composites on thermoplastic polyurethane (TPU) substrates. The SEC features two structural designs: straight-line and wavy-line configurations (Fig. 2e, f). Under an elongation of 3 mm, the straight-line and wavy-line structures exhibited resistance changes of 238.9% over 100 cycles and 243.6% over 200 cycles, respectively. The wavy-line configuration, with a smaller width-to-radius (w/r) ratio, demonstrated superior stretchability and sensitivity (33% resistance change per 1% strain), higher than the straight-line configuration (21% resistance change per 1% strain).
3. Creation of a metal network structure. Unlike the random arrangement of metal nanowires on flexible substrates, metal networks are generally well arranged, which is conducive to large-scale process production and results in low initial square resistances (as low as 0.12 Ω sq^−1^) [62–66]. For example, Chen et al. [65] developed a transparent Cu mesh SEC with good conductivity and multidirectional stretchability (Fig. 2g–i). The Cu mesh was initially prepared by template electroplating, followed by encapsulation with PDMS. The resulting SEC demonstrated a low sheet resistance of < 0.12 Ω sq^−1^ and could withstand a maximum strain of 160%. The resistance change remained below 5% under 60% strain. After 1000 cycles of stretching and releasing under 10% strain, the Cu mesh remained intact with negligible resistance change.

#### LM-Based SECs

LMs, such as eutectic gallium indium (EGaIn) and Galinstan, represent a kind of metal materials that exist in a liquid phase at or near room temperature, exhibiting characteristics of both fluids and metals [67, 68]. LMs possess high electrical conductivity, thermal conductivity, and chemical stability [69–72]. Given their inherent fluidity, LMs generally require integration with a supporting polymer matrix to form a reliable SEC for practical applications, and there have been mainly three integration strategies.

1. Injection of LMs into elastomer microchannels

**Fig. 3:**
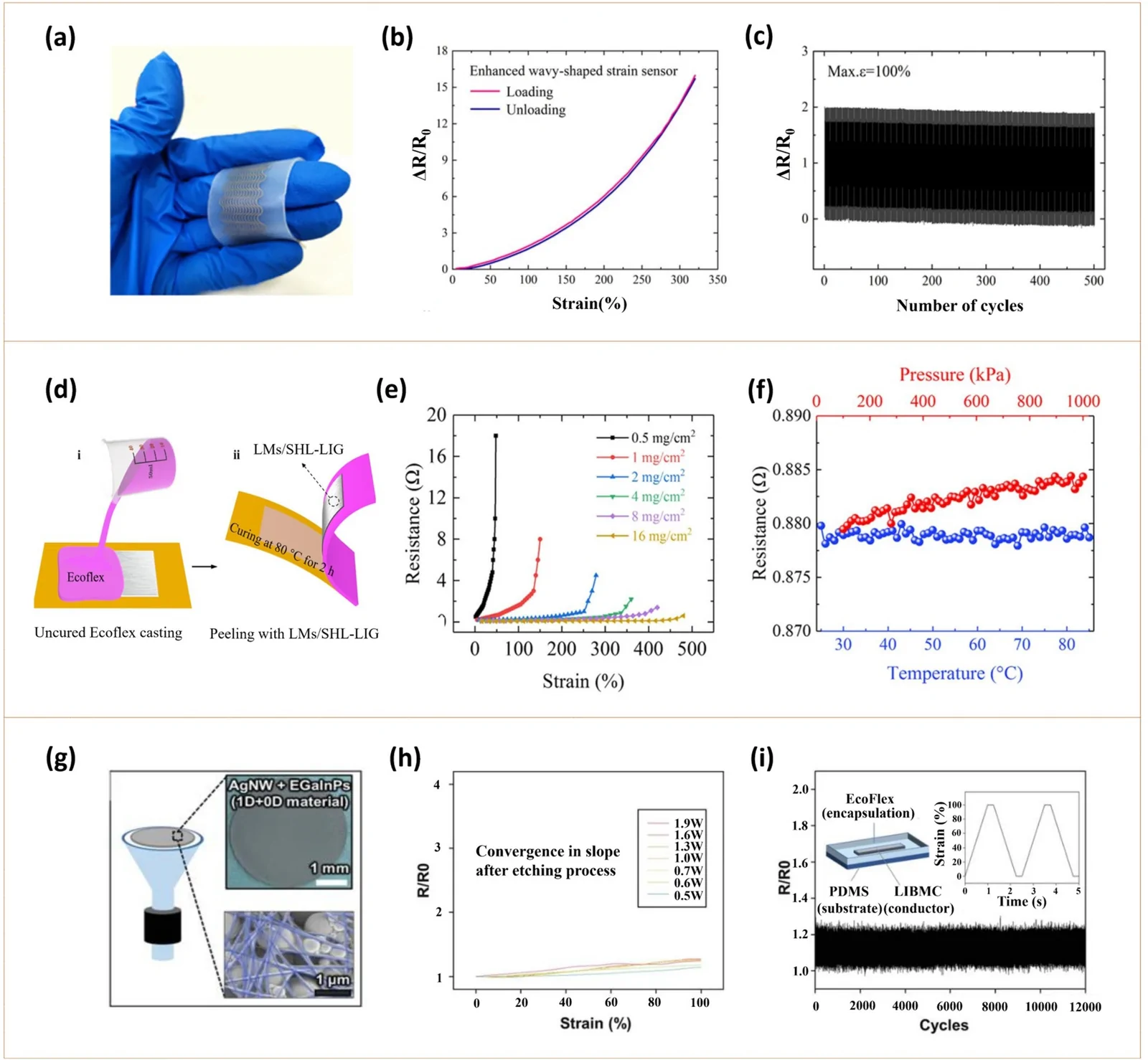
Liquid metal-based SECs. **a** Photograph of the LM-based SEC fabricated by injecting EGIn into a wavy microchannel Ecoflex. **b** Relative resistance changes of the SEC as a strain sensor when stretched from ε = 0 to 320%. **c** Δ*R*/*R*_0_ response of the SEC strain sensor over 500 stretching/releasing cycles with a strain of 100%. Reproduced with permission [77]. Copyright 2020, American Chemical Society. **d** Preparation of the SEC by transferring LMs/SHL-LIG patterns onto an Ecoflex substrate. **e** Resistance vs. strain measurements of the SEC under various mass loadings. **f** Resistance measurements of the SEC in the pressure range of 100–1,000 kPa and temperature range of 25–85 °C. Reproduced with permission [81]. Copyright 2023, American Chemical Society. **g** Schematic diagram, optical image, and pseudo-color SEM image of the AgNWs-EGaInPs SEC. **h** Relative resistance change as a function of uniaxial tensile strain of the LM/AgNWs SEC after etching with various laser power irradiations. **i** Relative resistance changes of the laser-irradiated biphasic metallic LM/AgNWs SEC subjected to cyclic uniaxial tensile loading to 100% for up to 12,000 cycles. The schematic illustration shows the structure of the LM/AgNWs SEC, and the inset describes the operating profile of the applied strain. Reproduced with permission [90]. Copyright 2022, Wiley‐VCH GmbH

The LM can be sealed in a soft elastomer by injection [73–77]. For example, Chen et al. [77] fabricated an SEC by injecting EGaIn into a wavy microchannel elastomer matrix (Fig. 3a–c). As a first step, the elastomer (Ecoflex) was poured into a microfluidic channel mold to solidify. A layer of elastomer was then spin coated on its surface. Finally, EGaIn was injected into the microchannel using a syringe. The fabricated SEC exhibited an increase in resistance with applied strain, with a relative resistance change (ΔR/R_0_) of approximately 2 at 100% tensile strain (R and R_0_ are the measured resistances under a certain strain and zero strain, respectively). The SEC was applied as a microfluidic flexible strain sensor that can withstand a strain of up to 320%, with Δ*R*/*R*_0_ versus strain curves exhibiting a monotonic increase with minor discrepancy. The SEC-based strain sensor shows stable performance (a tiny drift of 3.96%) under dynamic loading of 500 cycles of stretching/releasing at a peak strain of 100%.

(2) Adhesion and patterning of LMs onto elastomer surfaces

Various techniques, including 3D printing, molding, embossing transfer, and screen printing, have been applied to pattern LMs onto diverse elastomer substrates for the fabrication of SECs [78–81]. The introduction of metal NWs into LM followed by selective laser processing and etching can obtain self-supporting LM films, which can be applied directly to curved surfaces [82]. However, the high surface tension of LMs and their weak interfacial bonding with most elastomers still pose key challenges. To address these limitations, Wang et al. [81] proposed a method of combining LMs with soft elastomers using a super-hydrophilic laser-induced graphene (SHL-LIG) process (Fig. 3d–f). This involved coating a polyimide (PI) film with Cu to navigate LMs into specific patterns. The resulting LMs/SHL-LIG was then transferred to an Ecoflex substrate to obtain an SEC. This SEC exhibited a low sheet resistance of 3.54 mΩ sq^−1^ and could extend up to 480%. In addition, the resistance of this SEC changed by only 8% at 300% tensile strain and demonstrated strong insensitivity to temperature and pressure changes.

By inkjet printing, the LM acts as the printing ink in the preparation of SECs. Since the conductivity is limited by the formation of an insulating oxide layer outside the liquid metal particles (LMPs), the LM needs to be doped or modified [83–86]. For example, Veerapandian et al. [86] introduced hydrogen (H) doping on the surface of the LM oxide layer using ultrasonic treatment to enhance both conductivity and deformability. This H-doped LM solution was then employed as the ink for nozzle printing to manufacture circuit lines on a PDMS substrate. The metallic conductivity of the prepared printed circuit reached 25,000 S cm^−1^. Under 500% uniaxial stretching, the resistance of the circuit increased from 2.4 to 2.9 Ω.

(3) Self-assembly of modified LMs into films

The LM-based SECs can also be prepared by forming an LM film and then adhering it to an elastomer. To achieve spontaneous film formation, the LM needs to be modified to overcome the problem of high surface tension through techniques including the laser-induced method, thermal evaporation method, and solvent treatment method [87, 88].

The laser-induced method induces plasma resonance on the local surface of LM by laser irradiation, promotes the rupture of the oxide shell on the surface of LMPs, and enhances the interface adhesion between the LM and substrate, thus facilitating the spontaneous formation of LM films [89, 90]. Cho et al. [90] developed an SEC combining LM and AgNWs and regulated the degree of entanglement of these two-phase materials through a laser-induced photothermal reaction, enabling high-precision patternization and spatial programming of electromechanical properties in a single step (Fig. 3g–i). The obtained SEC achieved an electrical conductivity of 8.65 × 10^5^ S m^−1^, a relative resistance change of about 1.27 at 100% tensile strain, and maintained stable conductivity over 12,000 cycles at 100% strain.

The thermal evaporation method enhances the interfacial adhesion between the LM and the substrate by thermally vaporizing nanoclusters, such as indium (In)/gallium (Ga) nanoclusters, onto the substrate. Subsequent selective formation of an oxide layer in the air allows for the creation of a multi-layer LM network [91–93]. For example, Han et al. [93] proposed an SEC composed of an In/oxide film/Ga (InOG) structure. The InOG was obtained by depositing In nanoclusters onto an O_2_ plasma-treated TPU substrate using high-vacuum thermal evaporation. The sample was then exposed to air for a few seconds to form a thin layer of In oxide (In_2_O_3_)/In hydroxide on its surface, followed by the deposition of Ga nanoclusters onto the oxide layer via thermal evaporation. In the InOG structure, In and Ga were separated by an oxide film, which enhances the wettability of Ga, resulting in a multi-layer nanocluster network. The resistance of the InOG structure was reduced during the stretching process, which could be mainly attributed to two factors: 1) the increase in the size of the In and Ga nanoclusters leads to a decrease in sheet resistance, and 2) the fracturing of the interlayer oxide film during stretching initiates the formation of EGaIn and creates a new electrical pathway with the surrounding nanoclusters. After 50,000 fatigue tests at 50% tensile strain, the InOG’s resistance increased by no more than 50%.

The solvent treatment method represents an advanced technique for interface modification based on the selective interaction between a solvent and LM’s surface oxide. This method effectively removes the Ga oxide passivation layer on the surface of the LM droplet through the permeation of the solvent and reduces the thickness of the interfacial oxide layer to a nanometer level. In subsequent processing, the interdroplet oxide can be broken by mechanical stretching so that the LM can form a film on the substrate [94–96]. For example, Vallem et al. [96] reported an SEC based on LMPs where the LM surface was chemically modulated by ultrasonic crushing of EGaIn combined with solvent treatment. Specifically, hydrochloric acid and 1,6-hexane dithiol were added to an isopropyl alcohol solution containing LMPs for ultrasonic treatment. Subsequently, the interdroplet oxide was broken by stretching the substrate, enabling the formation of a film on the substrate. The prepared SEC exhibited high electrical conductivity (1.64 × 10^5^ S m^−1^), a large surface area (1,257% greater than LM film with the same location), and an almost strain-insensitive resistance (normalized resistance (*R*/*R*_0_) = 1.23 at 600% strain).

### Inorganic Nonmetallic Materials-Based SECs

Inorganic nonmetallic materials, including carbon nanomaterials, MXenes, and certain metal oxide semiconducting nanomaterials such as In_2_O_3_, have found applications in the preparation of SECs. Among them, carbon nanomaterials and MXenes, which offer high conductivity, good flexibility and contribute to the mechanical robustness of the SECs [97–100], will be the primary focus of this section.

#### Carbon Nanomaterials-Based SECs

Carbon nanomaterials can be divided into zero-, one-, two-, and three-dimensional (0D, 1D, 2D, and 3D) nanostructures. Among them, 1D and 2D carbon nanomaterials are more commonly applied in SECs due to their high electrical conductivity and flexibility [101, 102].

1. 1D carbon nanomaterials-based SECs

**Fig. 4:**
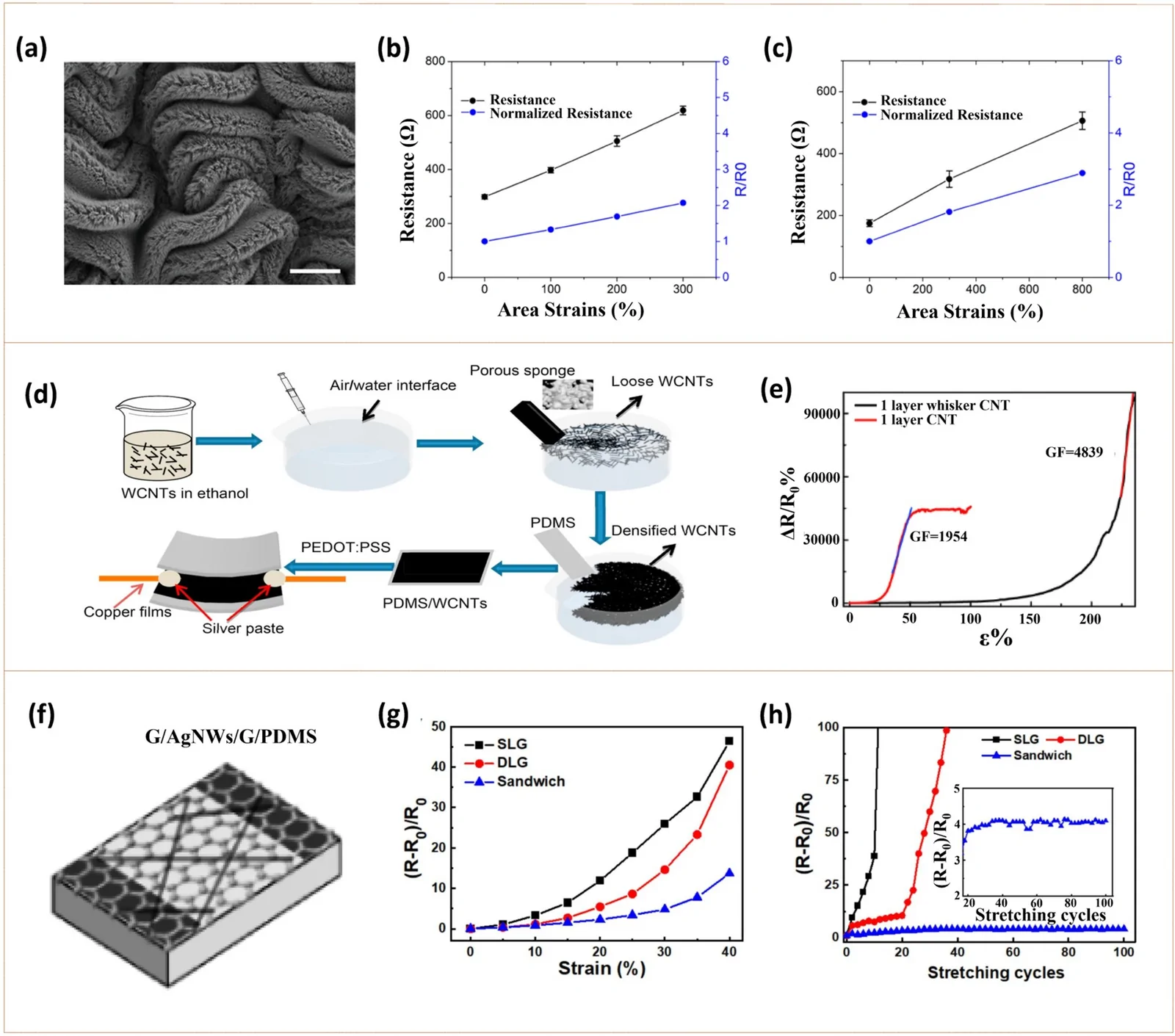
Carbon nanomaterials-based SECs. **a** SEM image of the crumpled pattern formed by the CNT-forest on a fully relaxed elastomer substrate (VHB 4910) with a pre-strain up to 300% × 300%. Scale bar, 100 µm. **b** Resistance variation of a uniaxial crumpled CNT SEC with a pre-strain up to 300%. **c** Resistance variation of a biaxially crumpled CNT SEC with a pre-strain up to 200% × 200%. Reproduced with permission [109]. Copyright 2019, WILEY‐VCH Verlag GmbH & Co. KGaA, Weinheim. **d** Schematic illustration of the fabrication of whisker-CNT (WCNT) nanocomposite films and the structure of the SEC. **e** The relative resistance changes of WCNT-based SECs and conventional CNTs-based SECs during stretching. Reproduced with permission [110]. Copyright 2021, Elsevier Ltd. **f** Schematic diagram of the G/AgNW/G sandwich structure. **g** Variation in electrical resistances with tensile strain for single-layer graphene (SLG), double-layer graphene (DLG) and the sandwich structure. **h** Stretching stability under 20% strain for the SLG, DLG and sandwich structure. Reproduced with permission [117]. Copyright 2021, MDPI

Carbon nanotubes (CNTs) and carbon nanofibers (CNFs) are typical 1D carbon nanomaterials. CNTs offer advantages such as high conductivity, large surface area, good flexibility, and high chemical stability [103–105]. However, the widespread adoption of CNTs in SECs is hampered by: (1) sheet resistance exceeding 100 Ω sq^−1^ due to impurities introduced during mass manufacturing and (2) limited stretchability of CNT fibers or films, leading to a rapid increase in resistance upon stretching. To improve the strain tolerance and conductivity of CNTs-based SECs, strategies such as network structure designs, binding CNTs with soft elastomers, and prestretch-release processes of CNT/elastomer composites are usually employed [106–110]. Cao et al. [109] developed a layered CNT SEC by transferring a crumpled vertically aligned CNT-forest onto an elastic substrate (VHB 4910) using a thermal annealing process in an atmospheric environment (Fig. 4a–c). The flexibility and intertwined networks within the crumpled CNT-forest allowed the film to maintain good conductivity throughout cyclic crumpling/unfolding, enabling the creation of stretchable and robust SECs that were applied as the electrodes for supercapacitors. Zhang et al. [110] proposed an SEC based on whisker-CNTs (Fig. 4d, e). The SEC was obtained using a simplified Langmuir–Blodgett method, where loose whisker-CNTs were densified through porous sponge capillary compression to form a conductive network, which was then laminated between PDMS elastic substrates. Its conductivity could reach 8,156 S m^−1^ and remained stable after 1,000 cycles at 40% strain.

(2) 2D carbon nanomaterials-based SECs

Graphene is a 2D form of carbon atoms packed in a hexagonal lattice, with unique properties such as a theoretically large specific surface area of 2,630 m^2^ g^−1^, high carrier mobility up to 200,000 cm^2^ V^−1^ s^−1^, high chemical/thermal stability, and high flexibility [111–113]. While graphene itself is not inherently stretchable, graphene-based SECs are typically prepared by compounding graphene with other materials. For example, graphene can form a multi-layer structure with nanomaterials possessing a high aspect ratio to manufacture SECs [114–117]. Huang et al. [117] inserted AgNWs between two graphene layers to form a G/AgNWs/G sandwich structure as an SEC (Fig. 4f–h). The AgNWs not only suppress the formation of cracks and pores in the graphene layers, which could result in conductivity loss under tensile strain, but also bridge existing cracks to compensate for the conductive path loss. Compared with one layer and two layers of graphene, the G/AgNWs/G sandwich structure exhibited the slowest rate of resistance change under strain. The conductivity of the sandwich structure remained stable after 100 stretching/releasing cycles under 20% strain.

Graphene-based SECs can also be prepared by coating or transferring graphene onto a pre-stretched elastomer layer [118–121]. For example, Lin et al. [121] reported an SEC based on pleated graphene. This involved initially transferring multiple layers of graphene from Cu foil to a PDMS supporting layer and subsequently onto a pre-stretched acrylic elastomer film. Upon release of the pre-stretch, the graphene formed a pleated structure due to compression. The transfer of the pleated graphene to PDMS was facilitated by the differential swell ratio between the solvent (acetone) swollen elastomer and the target substrate. In the 0–75% strain range, the resistance demonstrated a linear change with strain, with a sensitivity coefficient of 0.557. The stability was maintained after 2,000 cycles at 40% strain, and the elongation at break reached 150%.

#### MXenes-Based SECs

MXenes are a class of 2D metal carbides, nitrides, and carbon nitrides. The chemical formula of MXenes is M_n + 1_X_n_T_x_ (*n* = 1 ~ 4), where M represents early transition metals of group Ⅲ–Ⅵ, such as Ti, Zr, V, and Mo, X denotes carbon atoms or nitrogen atoms, n indicates the number of layers of carbon or nitrogen, and T_x_ means the surface groups on the outermost M layer, typically –OH, –O, –F, and –Cl. MXenes exhibit unique properties including high electrical conductivity, large specific surface area, good mechanical properties, and good hydrophilicity, making them promising candidates for SECs [122–125].

**Fig. 5:**
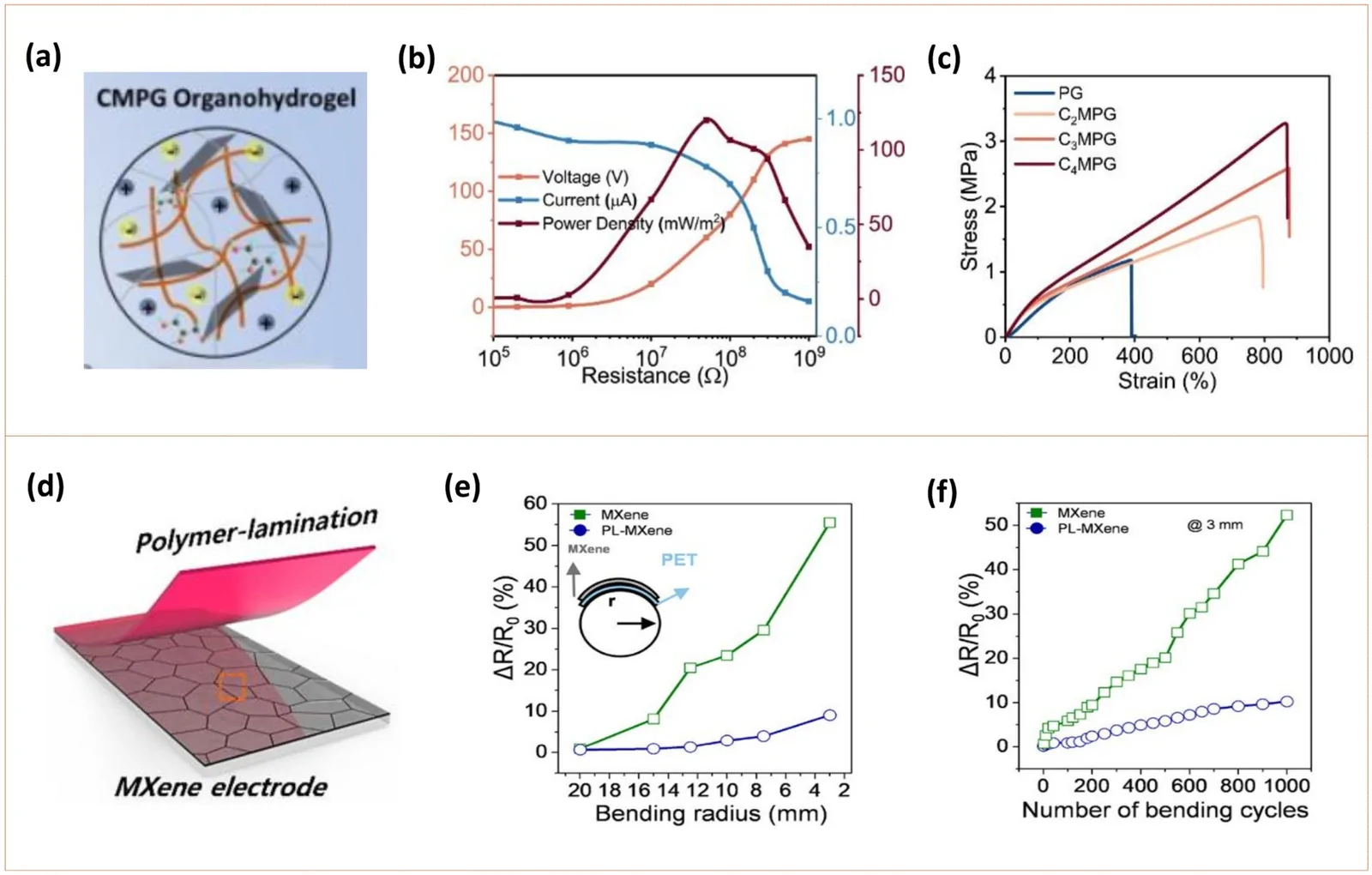
MXenes-based SECs. **a** Schematic diagram showing the internal structure of the cellulose and MXene enhanced PVA organic hydrogel. **b** Voltage, current, and output power of the TENG based on the MXene-based SEC at different external load resistances. **c** The tensile stress–strain curves of MXene-based SEC with different CNF contents. Reproduced with permission [129]. Copyright 2023, Elsevier B.V. **d** Schematic illustration of the PL-MXene-based SEC composed of MXene flakes with a protective poly(4-vinylphenol) layer. Δ*R*/*R*_0_ of the MXene and PL-MXene SEC on PET substrates with respect to the **e** bending radius and **f** number of bending cycles (bending radius: 3 mm). Reproduced with permission [134]. Copyright 2021, American Chemical Society

Among the MXene family, Ti_3_C_2_T_x_ is the most extensively studied for SEC applications, and the preparation methods for MXene-based SECs are similar to those used for graphene-based SECs [126–134]. For example, Zhou et al. [129] prepared a freeze-resistant and mechanically strong polyvinyl alcohol (PVA) organic hydrogel SEC by integrating 1D CNF with 2D MXene (Fig. 5a–c). Providing high conductivity through molecular interactions and geometric synergy, glycerol and KOH were also incorporated to improve the stretching and freezing resistance of the hydrogel. The prepared SEC maintained a conductivity of 6.2 S m^−1^ at − 20 °C and exhibited an elongation at break of up to 866%. Li et al. [134] coated a thin layer of poly(4-vinylphenol) on an MXene layer using a two-step spinning coating method and obtained a polymer-laminated-MXene (PL-MXene) SEC (Fig. 5d–f). An electroluminescent display prepared using the PL-MXene SEC functioned normally under high temperature (70 °C) and humidity (50%) conditions and exhibited excellent antioxidant properties. In addition, the SEC maintained good transparency, with a transmittance of approximately 76% at a wavelength of 550 nm.

### Conductive Polymer-Based SECs

Conductive polymers have shown great potential for the preparation of SECs owing to their inherent elasticity and flexibility, along with the tunability of polymer chain interactions and chemistry [135–140]. These polymers, typically *π*-conjugated systems, exhibit inherent electrical conductivity arising from the delocalized *π* electrons that can move freely throughout the polymer chain. While conductive polymers generally possess some degree of stretchability, their tensile properties can be further improved through strategies such as the addition of small molecule plasticizers and solution treatment. Common conductive polymers include polypyrrole (PPy), polyaniline (PANI), poly (3,4-vinyldioxythiophene):poly (styrene sulfonic acid) (PEDOT:PSS). Among them, PEDOT:PSS is the most studied for SECs [141, 142]. PEDOT:PSS can form self-supporting films and is non-toxic and chemically adjustable (allowing covalent bonding with biomolecules), although its tensile properties are somewhat limited. The following methods have been applied to improve its tensile properties and/or electrical conductivity.

**Fig. 6:**
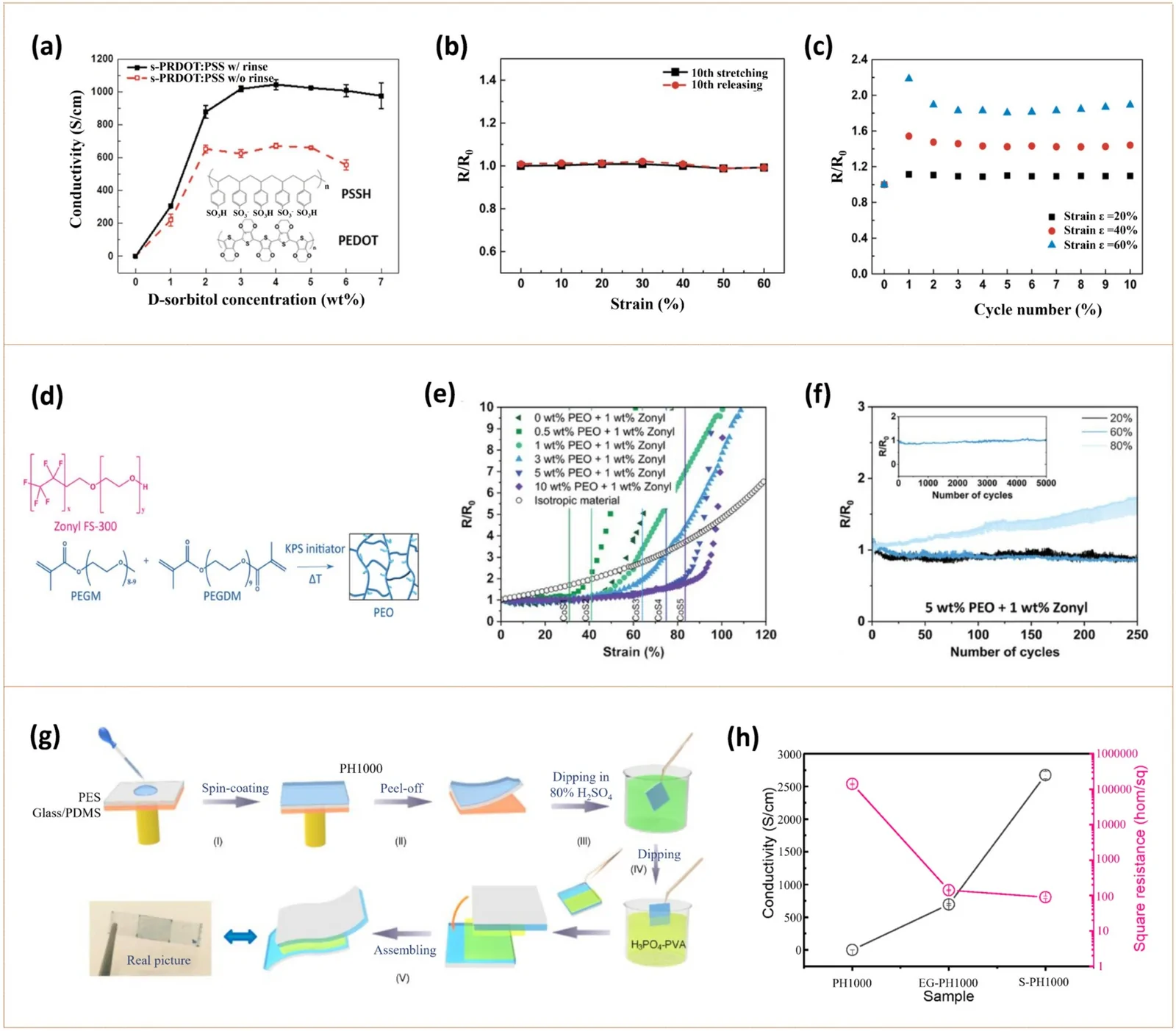
Conductive polymer-based SECs. **a** Variation in the conductivity of s-PEDOT:PSS SECs before and after water rinsing with different concentrations of d-sorbitol in the mixture solution. **b** Variation in the normalized resistance of a 6 wt% s-PEDOT:PSS SEC with post-water rinsing at the 10th stretching/releasing cycle. *R*_0_ is the resistance of the polymer film in the relaxed state after the 9th cycle. **c** Variations in the normalized resistance of a 6 wt% s-PEDOT:PSS SEC with post-water rinsing during stretching/releasing cycles under maximum strains of 20, 40, and 60%. Reproduced with permission [146] Copyright 2019, American Chemical Society. **d** Chemical structure of the surfactant Zonyl and the compounds used to form the PEO polymer network. **e** Relative resistances of the PEDOT:PSS SEC with various wt% of PEO and 1 wt% Zony under strain. **f** PEDOT:PSS SEC containing 5 wt% PEO and 1 wt% Zonyl under different strains. The inset shows the resistance behavior over 5,000 cycles at 60% strain. Reproduced with permission [150]. Copyright 2020, WILEY–VCH Verlag GmbH & Co. KGaA, Weinheim. **g** Schematic diagrams for the preparation of the S-PH1000 (an SEC based on PEDOT:PSS immersed in an 80 wt% H_2_SO_4_ solution) and flexible supercapacitor based on the SEC. **h** Conductivity and square resistance of the PH1000, EG-PH1000 (5 wt% ethylene glycol-doped PH1000) and S-PH1000 films. Reproduced with permission [155]. Copyright 2020, DMPI

1. Incorporation of small molecule plasticizers [143–146]. The insertion of a small molecule plasticizer into the PEDOT:PSS chain can weaken the strong H bond and electrostatic interaction in the PSS phase, thereby reducing the rigid binding between molecular chains. At the same time, the plasticizer, as a lubricating medium, promotes the slippage and rearrangement of molecular chains, disperses the stress concentration, and thus improves the tensile properties of the material. For example, He et al. [146] prepared a D-sorbitol-PEDOT:PSS (s-PEDOT:PSS) SEC by spin coating a mixed solution of PEDOT:PSS and D-sorbitol on a glass substrate (Fig. 6a–c). The conductivity and tensile properties of PEDOT:PSS were improved by adding the biocompatible D-sorbitol. The prepared PEDOT:PSS SEC exhibited a conductivity of up to 1,000 S cm^−1^ at a tensile strain of 60%, with negligible change in conductivity after 10 stretching/releasing cycles. The enhanced performance was due to the disruption of H bonds between the PSSH chains by D-sorbitol, making the PSSH chains more prone to conformational changes under stress.
2. Surfactant treatment [147–150]. Surfactants are embedded into the PSS phase via their fluorinated hydrophobic chains in their amphiphilic structures, partially shielding the strong H bonds and electrostatic interactions between PSS chains. This reduces the rigid binding of molecular chains and promotes phase separation between PEDOT and PSS, leading to the formation of a more continuous flexible network. Dauzon et al. [150] treated PEDOT:PSS with a mixed solution containing polyethylene oxide (PEO) as a precursor, Zonyl surfactant, and 5% dimethyl sulfoxide (DMSO) as a solvent, resulting in a transparent PEDOT:PSS-based SEC (Fig. 6d–f). The obtained SEC exhibited a conductivity of up to 1,230 S cm^−1^. The *R*/*R*_0_ of the conductor with 5 wt% PEO + 1 wt% Zonyl increased by 1.7 times after 250 cycles at 60% strain.
3. Doping. The conductivity of PEDOT:PSS can be improved by doping polar solvents, strong acids, ionic liquids, and other substances. Polar solvents can induce a solvation effect, bringing the PEDOT chains closer together, which is conducive to the transmission of electrons. Strong acids can impose a protonation effect, promote the rearrangement and accumulation of PEDOT chains, and form more orderly conductive channels. The introduction of ionic liquids can provide additional ion transport channels and increase the overall conductivity [151–155]. For example, Song et al. [155] spin-coated a PEDOT:PSS aqueous solution on a polyether sulfonate (PES) substrate and then treated it with 80 wt% H_2_SO_4_. Following with a post-processing, an SEC with a maximum conductivity of 2,673 S cm^−1^, high transparency (> 85%), and a sheet resistance of 89 Ω sq^−1^ was obtained (Fig. 6g, h).

### Composite Materials-Based SECs

The composite materials-based SECs reviewed in this section mainly focus on those manufactured by blending stretchable polymers with conductive fillers. The stretchable polymers serve as the supporting matrix, while the conductive fillers are dispersed within this matrix to form conductive pathways. The increase in the volume fraction of the conductive network will generally increase the conductivity of the composite materials-based SECs. When the volume fraction exceeds a certain value, the conductivity of the composite materials-based SECs will reach what is called the percolation threshold. At present, the general effective medium (GEM) model is commonly employed to study the electrical performance trend of the composite materials-based SECs [156–159]. The model formula is as follows [156]:

1 $$\sum\limits_{1}^{N} {\varphi_{i} \frac{{\left( {_{{\mathrm{i}}}^{\frac{1}{t}} - \sigma_{m}^{\frac{1}{t}} } \right)}}{{\sigma_{{\mathrm{i}}}^{\frac{1}{t}} + A\sigma_{m}^{\frac{1}{t}} }}} = 0$$

2 $$A = \frac{{1 - \varphi_{c} }}{{\varphi_{c} }}$$

where *σ*_*m*_ is the conductivity of the stretchable composite materials-based SECs, *σ*_*i*_ is the conductivity of the i-th component, *φ*_*i*_ is the volume fraction of the i-th component, *φ*_*c*_ is the percolation threshold, *t* is the critical index, and *A* is a parameter that changes with the percolation threshold. In this model, the critical exponent *t* can be determined through calculation or curve fitting techniques.

**Fig. 7:**
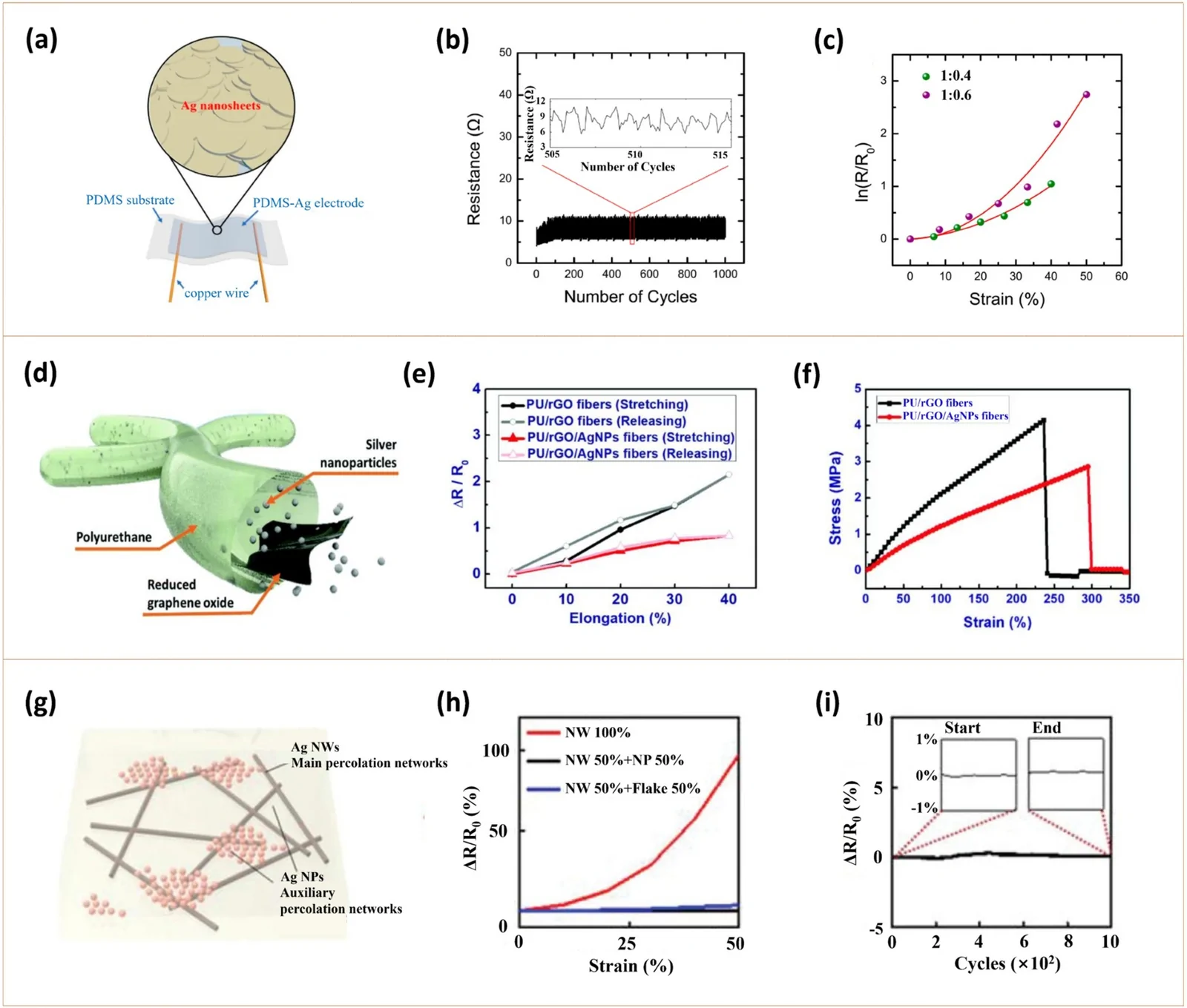
Composite materials-based SECs. **a** Schematic illustration of the SEC based on PDMS-Ag nanosheets composite. **b** Resistance of the SEC under 1,000 stretching/releasing cycles at a strain of 20%. **c** The tunneling effect theory is employed to fit the resistance variations in SECs (mass ratios of silver adhesive to PDMS are 1:0.4 and 1:0.6) during the stretching process within a strain range of 0–50%. Reproduced with permission [163]. Copyright 2022, DMPI. **d** Schematic diagram of a patterned transparent reticular nanofiber SEC based on the PU/rGO/AgNPs composite. **e** Δ*R*/*R*_0_ vs. elongation of the PU/rGO and PU/rGO/AgNPs SECs on PDMS substrates. **f** Stress–strain curves of the PU/rGO and PU/rGO/AgNPs SECs. Reproduced with permission [175]. Copyright 2019, Royal Society of Chemistry. **g** Schematic illustration of the percolation networks comprising Ag NWs and Ag NPs. **h** Relative resistance changes of the SECs based on 0D, 1D, 2D Ag nanomaterials, and SEBS composite under a strain range of 0–50%. **i** Relative resistance changes of the SEC based on 40 wt.% Ag NWs, 40 wt.% Ag NPs and 20 wt.% SEBS during 1,000 stretching/releasing cycles with 50% uniaxial strain. The insets show magnified views of the relative resistance changes at the beginning and end of the cycles. Reproduced with permission [176]. Copyright 2022, Wiley‐VCH GmbH

Composite materials-based SECs have been prepared by mixing polymers with metal nanostructures [160–163], inorganic nonmetallic materials [164–166], conductive polymers [167–169], and a combination of different conductive fillers [170, 171]. Li et al. [163] reported an SEC with a low resistance and stable performance based on a PDMS-Ag nanosheet composite (Fig. 7a–c). With the synergistic action of the high tensile properties of PDMS and the excellent electrical conductivity of Ag nanosheets, the minimum resistivity of the SEC reached 4.28 Ω m, and the resistance increased by about 4 Ω under 20% strain. Although the internal conductive pathways were damaged at 200% strain, the SEC still worked normally after the stress was released. Dong et al. [166] prepared an SEC using PU as the matrix and CNT as the conductive filler. The dynamic boron ester bonds and H bonds in PU endowed the electronic conductor with a self-healing efficiency of 78%, a tensile strength of 15.4 MPa, and an elongation at break of 420%, while the CNT contributed to a high conductivity of 0.57 mS cm^−1^. Kim et al. [169] blended PEDOT:PSS with a highly stretchable non-ionic waterborne PU (WPU) and coated the mixture onto a TPU film. WPU interacts with PEDOT:PSS through H bonding and coulomb attraction. By varying the WPU content, the electrical and tensile properties of the SEC could be tuned. At a WPU concentration of 2.0 wt%, the sheet resistance was about 400 Ω sq^−1^ and remained almost unchanged at 100% strain. Even at 400% strain, the surface of the SEC showed no signs of damage.

In order to further improve the conductivity and mechanical stability of composite materials-based SECs, a combination of various conductive fillers can be incorporated by leveraging the attractive forces between different fillers [172–175]. For example, Chio et al. [175] designed a stretchable transparent nanofiber network SEC (STNNE) based on an electrospun stretchable nanofiber network structure composed of a mixture of PU/reduced graphene oxide (rGO)/Ag nanoparticles (AgNPs) (Fig. 7d–f). The resistance of the STNNE film reached 210 Ω sq^−1^, with a mechanical stretchability of up to 40% and relatively high electrical stability.

Furthermore, the combination of surface-modified nanomaterials of different sizes can allow the composite materials-based SEC to maintain a percolation network under different strain levels, thereby improving both the tensile properties and electrical conductivity of the SEC [176]. For example, Jung et al. [176] employed an optimum combination of 0D, 1D, and 2D Ag nanomaterials treated with 1-decanethiol to form an SEC that demonstrated insensitivity to uniaxial or biaxial strain (Fig. 7g–i). The surface modification of Ag nanomaterials by 1-decanethiol promoted the strain-induced rearrangement of Ag nanomaterials in a viscoelastic matrix (poly(styrene ethylene butylene styrene), SEBS), which helped preserve a connected percolation network under strain. This SEC, composed of diverse dimensional Ag nanomaterials and block copolymer elastomers, exhibited highly stable electrical properties with less than 1% resistance change under less than 50% strain, and its initial conductivity reached 31,000 S cm^−1^.

In addition, through the dual-ligand surface-modified nanomaterials combined with high-humidity-environment control, the local bonding structure of the nano-network can be constructed to cooperatively enhance both the electrical conductivity and tensile strength of the composite materials-based SECs [177]. Jung et al. [177] modified the surface of AgNWs with a dual ligand of 1-propyl mercaptan and 1-decyl mercaptan. These modified AgNWs were then mixed with SEBS under highly humid conditions. The high humidity promoted local binding among the modified AgNWs. This localized binding improved the conductivity of the AgNWs network and strengthened the interconnections between AgNWs. The obtained SECs demonstrated excellent electrical conductivity (122,120 S cm^−1^) and stretchability (elongation at break reaching 200%). At 100% tensile strain, ΔR/R_0_ was approximately 5.

## Summary of This Chapter

SECs can be divided into metal-based, inorganic nonmetallic materials-based, conductive polymer-based, and composite materials-based SECs, based on their primary conductive components. Among metal-based SECs, solid metals (e.g., Ag) are generally processed into nanostructures, which exhibit excellent electrical conductivity but lack sufficient flexibility. Coordination between conductivity and tensile properties for solid metal-based SECs can be achieved through methods such as micro-crack network regulations, hybrid structures incorporating metal nanowire interlayers, and designs like template electroplated metal mesh. LMs (e.g., EGaIn) exploit their fluidic properties combined with elastomer packaging strategies to construct highly stable conductive networks through means such as microchannel injection, adhesion and patterning of LMs on elastomer surfaces, and self-assembly of modified LMs into films. The high surface tension and weak interfacial bonding with most elastomers of LMs are the main challenges for LM-based SECs. Inorganic nonmetallic materials-based SECs mainly include carbon nanomaterials (e.g., CNT and graphene)- and MXenes (e.g., Ti_3_C_2_T_x_)-based SECs. Their mechanical adaptability can be enhanced through techniques such as coating on pre-stretched elastomers and combination with other nanomaterials to form multi-layer structures. Conductive polymer-based SECs are predominantly based on PEDOT:PSS, whose stretchability can be enhanced by incorporating of small molecule plasticizers and surfactant treatment, and conductivity can be improved by doping with substances such as polar solvents, strong acids, and ionic liquids. The addition of small molecule plasticizers and surfactants enhances the stretchability of PEDOT:PSS by weakening the strong H bond and electrostatic interaction in the PSS phase, while the doping of polar solvents, strong acids, and ionic liquids brings the PEDOT chains closer, facilitating electron transfer and thus improving conductivity. Composite materials-based SECs are typically manufactured from a mixture of stretchable polymers and conductive fillers. The conduction–stretchability synergies can be enhanced through methods like multi-packing coordination, nano-size regulation, and dual-ligand surface modification. The GEM model can be applied to optimize the percolation threshold to analyze the optimal performance of the conductive network. Table 1 summarizes the typical characteristics of SECs categorized on the basis of different conductive materials.

**Table 1:** Typical characteristics of SECs categorized on the basis of different conductive materials

Type of SECs	Conductive materials	Materials	Conductivity or sheet resistance	Substrate or supporting matrix	Stretchability	Δ*R*/*R*_0_ under strain	Preparation Process	Reference
Metal-based SECs	Solid metal	AgNPs	–	PDMS	200%	4.1 under 100% strain	Laser deposition	[54]
		AgNPs	8 ~ 9 Ω sq^−1^	Parylene	50%	–	Vacuum vapor deposition template method	[55]
		AgNPs	–	PDMS	100%	1.4 under 30% strain	Screen printing and sacrificial	[56]
	Liquid metal	EGaIn	22.532 S cm^−1^	TPU	2260%	1.59 under 1650% strain	3D printing	[76]
		EGaIn	–	PVA hydrogel	400%	0.8 under 100% strain	Noncontact laser cutting and magnetic coating	[78]
		EGaIn	8.65 × 10^5^ S m^−1^	PDMS, PET, PI	100%	1.27 under 100% strain	Laser sintering	[90]
Inorganic nonmetallic materials-based SECs	Carbon nanomaterials	Whisker CNTs	8156 S m^−1^	PDMS	420%	–	Langmuir–Blodgett method	[110]
		Graphene	7 S cm^−1^	PDMS	80%	0.244 under 80% strain	Chemical vapor deposition	[116]
		Laser-induced graphene	114 Ω sq^−1^	SEBS	300%	0.32 under 5% strain and 3.75 under 30% strain	Laser sintering	[118]
	MXenes .	Ti_3_C_2_T_x_	2.3 S cm^−1^	PTFE	180%	–	Dot-matrix drop-casting method	[127]
		Ti_3_C_2_T_x_	8.7 S m^−1^	PVA	820%	–	Chemical vapor deposition	[129]
Conductive polymer-based SECs	Conducting polymers	PEDOE:PSS /PR^a^	1603 S cm^⁻1^	SEBS/silicon	–	3.4 under 100% strain	Drop casting	[143]
		PEDOT:PSS/CNP^*b*^	1230 S cm^−1^	PDMS	120%	–	Drop casting	[147]
Composite materials-based SECs	Composite conductive materials	PU/rGO/AgNPs	48 S cm^−1^	PDMS	–	0.83 under 40% strain	Electrostatic spinning	[175]
		Ag NWs/SEBS	31.000 S cm^−1^	–	800%	0.01 under 50% strain	Solution mixing	[176]

^a^Polyrotaxane

^b^Cellulose nanofibril paper

## Structure Designs of SECs

The stretchability of SECs can be achieved through ingenious structural designs. Specific designs can circumvent the typical decline in conductivity observed under tensile strain, allowing the SECs to maintain their original conductivity within a certain strain range. The structure design of SECs can be categorized into three types based on the number of dimensions of stretchable directions: 1D, 2D, and 3D stretchable configurations.

### 1D Stretchable Structure Designs

1D stretchable structure designs can maintain the conductivity of the SECs during the stretching process in a single linear direction. This is primarily realized via buckling, spiral, wave, and kirigami structures [178–181].

1. Buckling structure

**Fig. 8:**
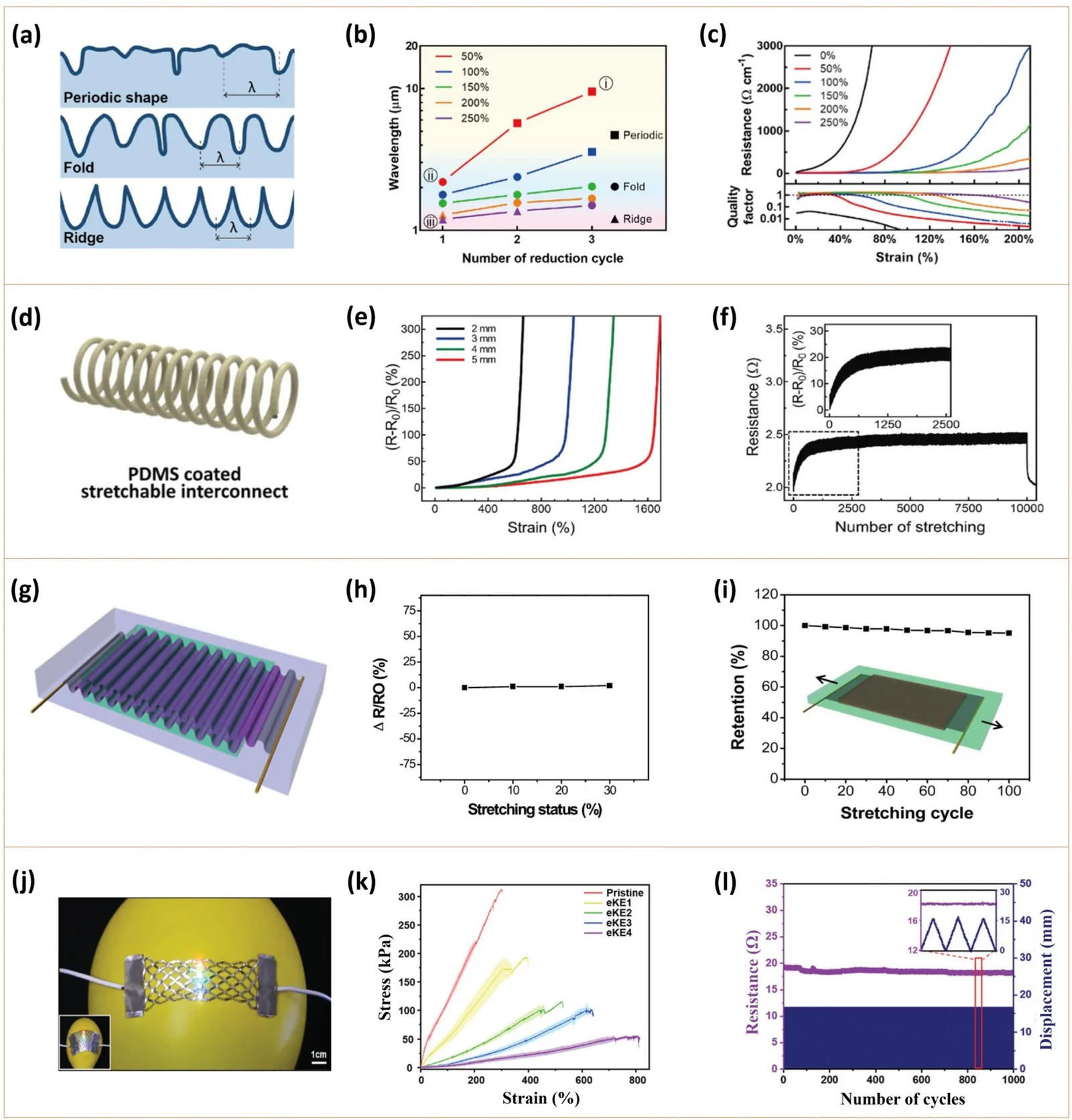
1D stretchable structure designs. **a** Schematic illustrations of the formed buckle shapes. **b** Wavelengths and shapes of buckled structures of the fibers based on the number of reduction cycles and pre-strain. **c** Resistance and quality changes of the buckled AgNPs/PU fibers (one reduction cycle) due to the applied tensile strain. Reproduced with permission [185]. Copyright 2023, American Chemical Society. **d** Schematic diagram of the helical fiber interconnect. **e** Normalized relative resistance changes of helical fibers with different helical diameters as a function of tensile strain. **f** Resistances of the PDMS-coated helical fiber with a helical diameter of 3 mm under 10,000 stretching/releasing cycles from 0 to 100% applied strain with a frequency of 0.5 Hz. The inset shows the range of stretching cycle numbers between 0 and 2,500. Reproduced with permission [42]. Copyright 2020, WILEY‐VCH Verlag GmbH & Co. KGaA, Weinheim. **g** Schematic diagram of a supercapacitor composed of wavy SECs. **h** Electrical resistance variation of the wavy shaped porous graphene as a function of the stretching status. **i** The capacitance retention of the supercapacitor over 100 cycles of stretching/releasing tests at 20% strain. The inset schematically illustrates the SECs-based supercapacitor in a stretched state. Reproduced with permission [194]. Copyright 2014, Royal Society of Chemistry. **j** Photograph of the LM-based elastic kirigami SEC. **k** Stress–strain curves of five different patterned SECs. **l** Cyclic loading test of the SEC for 1,000 cycles at 0–100% strain. Reproduced with permission [199]. Copyright 2023, Wiley‐VCH GmbH

1D buckling structures are mainly manifested as bending or torsion deformation along their longitudinal axis. These structures can be constructed by forming controllable folds on an elastic substrate, such as PDMS, through a prestretch-release process. In addition, the matrix can be precisely induced to form buckling morphologies of different characteristic scales by coordinating the gradient combination of pre-stretching amplitude and chemical reduction frequency [182–185]. Yoon et al. [185] applied a dense distribution of AgNP networks in PU to fabricate a strain-insensitive fiber conductor consisting of a highly conductive buckling shell through a simple chemical process (Fig. 8a–c). Repeated absorption and reduction in the Ag precursor increased the AgNP content within the fiber. By changing the number of absorption and reduction cycles, three kinds of buckling structures, meaning periodic (squares), fold (circles), and ridge (triangles) fibers, were created. With increasing chemical reduction cycles and decreasing pre-strain, the surface morphology of the fiber tended to transition from a ridge shape to a periodic shape. Finally, the experimental results showed that the ridge-shaped fibers exhibited lower resistances and superior tensile insensitivity. The critical strain gradually increased from 30 to 180% as the pre-strain went up from 50 to 250%.

(2) Spiral structure

When the spiral structure is stretched, the fibers inside will gradually straighten, similar to a spring. The inclined winding fibers progressively align with the direction of the applied tension, and the distance between adjacent coils increases, analogous to straightening a coiled telephone cord where the coil spacing widens and the overall length grows. The amplitude of the geometry adapts to accommodate the applied stress, allowing the SEC to be stretched without stress concentration in the material itself [42, 186–190]. Woo et al. [42] developed an SEC based on a highly stretchable spiral-structured PU-based fiber containing AgNPs with invariantly high conductivity (Fig. 8d–f). To impart electrical conductivity to the fibers, the Ag precursor solution was reabsorbed and reduced on the PU-based fibers. When the spiral diameter of the SEC was 3 mm, the resistance showed a negligible increase even at 1,000% strain. After 10,000 tensile cycles, the electrical properties remained stable. Liang et al. [189] constructed a stretchable PEDOT@bacterial cellulose (BC)/CNT hybrid spiral fiber with a “reinforced cement–sand” structure using a wet spinning and winding process. Dissolved BC acted as the bonding matrix, undissolved bacterial nanofibers and CNTs served as the supporting body, and PEDOT functioned as the reinforcing material. This structure avoids the reliance on an elastic matrix or auxiliary materials to provide elasticity. The spiral fiber exhibited elongation at a break of 1,175% and demonstrated good cyclic stability. Ma et al. [190] created shape-programmable LM fibers via the phase transition of Ga. The solid Ga wire could be easily molded into a spiral structure, and after coating the wire with PU, the structure was retained even after the Ga metal was liquefied. Spiral LM fibers fractured at approximately 1,273% strain, significantly higher than the fracture strain of 1D linear LM fibers at 358%.

(3) Wave structure

When the material of the wavy structure is stretched, the angle between adjacent elements gradually expands, and the distance between them widens synchronously. This dynamic adjustment mechanism effectively disperses and offsets the tensile stress, endowing the structure with sufficient stretchability [191–195]. Xie et al. [194] introduced a wavy PANI/graphene-based SEC. Initially, nickel (Ni) foam was manually manufactured into a wavy shape, followed by the growth of porous graphene on the wavy Ni foam using the atmospheric pressure chemical vapor deposition (CVD) method (Fig. 8g–i). The Ni skeleton was then removed by wet etching with a 3 M HCl solution. Finally, PANI was uniformly and densely deposited on the surface of graphene via pulse electrodeposition to obtain the PANI/graphene-based SEC. A supercapacitor composed of this SEC maintained high mechanical strength and capacitance at even 30% tensile strain. Yu et al. [195] prepared a wavy SEC, which was fabricated via bulk silicon micromachining and subsequently deposited perylene C on the wafer through thermal evaporation, followed by sputtering platinum onto it. The perylene C served as a flexible substrate to support the platinum. A capacitive sensor fabricated by embedding the wavy SEC into a PDMS layer exhibited low hysteresis (0.64%) and high sensitivity (a gauge factor of 0.27 at 25% strain).

(4) Kirigami structure

The kirigami structure is prepared by cutting materials into specially designed patterns, which effectively releases the internal stress of the material through out-of-plane deformation and enables the material to maintain stable electrical properties under large deformations. Similar to other structure designs, while the kirigami structure was able to increase the tensile limit of the electronic conductor to varying degrees, the inherent rigidity of the material still limits its ultimate elongation at break [196–199]. Choi et al. [199] developed an LM-based elastic kirigami SEC through a fusion of kirigami structural mechanics, an elastic silicone substrate, and an LM conductive material (Fig. 8j–l). The SEC was based on a silicone elastic film (Dragon Skin 10, thickness 500 μm) cut into a kirigami pattern using a laser, with its conductive layer constructed by magnetron sputtering deposition of a 10-nm-thick Au film and then coating with EGaIn. As an intermediate layer, the Au film not only enhanced the interface bonding force between the LM and the substrate but also effectively maintained the conductive stability of the electrode under extreme deformation by forming a solid–liquid biphase metal layer with EGaIn. The resistance of this SEC increased by 0.33 times under 820% tensile strain, showing good electromechanical stability.

### 2D Stretchable Structure Designs

2D stretchable structures can retain a certain conductivity when stretched along both the horizontal and vertical axes. The primary 2D stretchable structure designs for SECs include the buckling structure, snakeskin structure, and mesh structure [200–203].

**Fig. 9:**
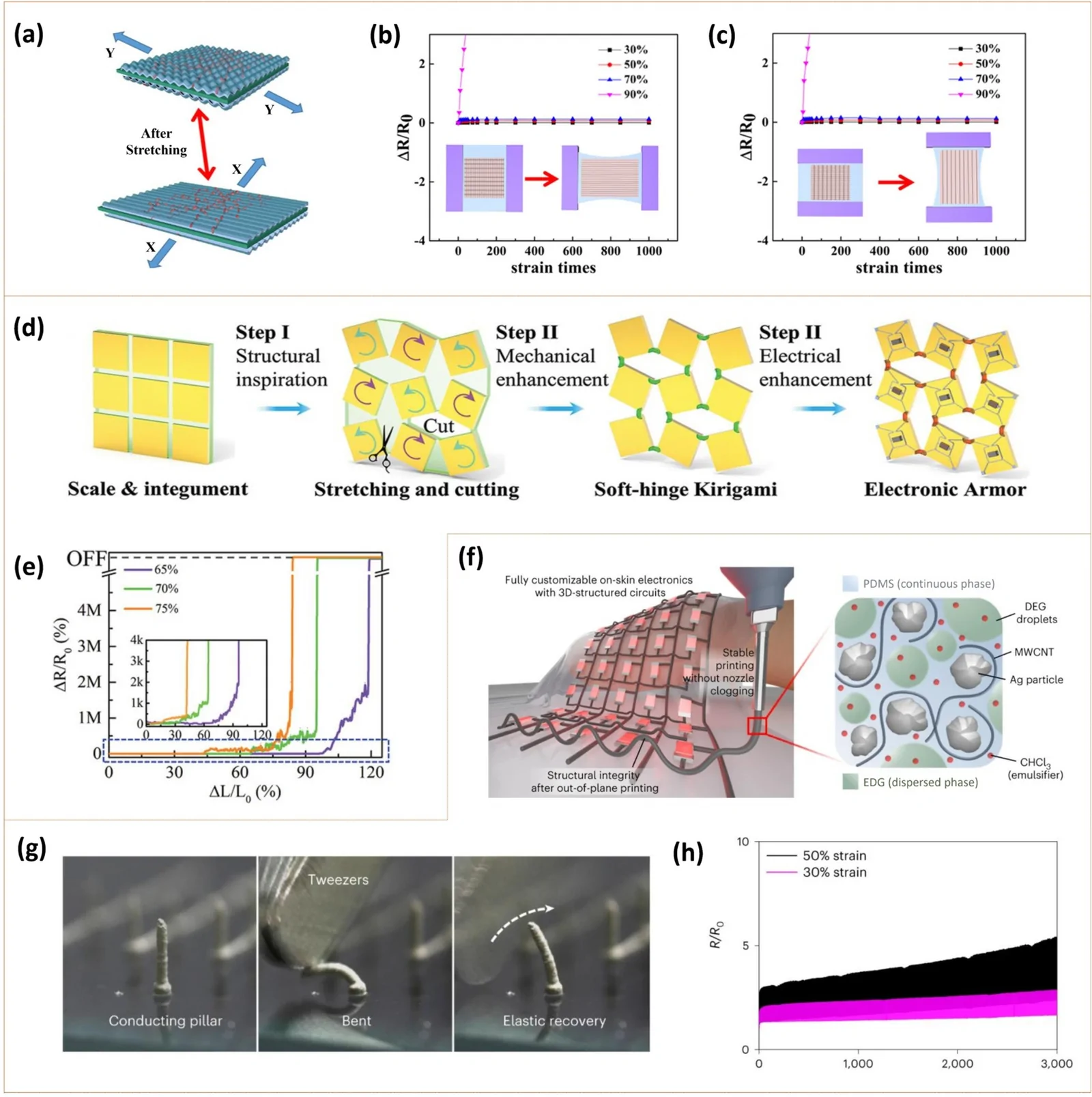
2D and 3D stretchable structure designs. **a** Schematic diagram of a sensor composed of SECs with 2D buckling structures. The relative resistance changes of the multidirectional SEC under cyclic stretching/releasing at different strains in directions of **b** *x*-axis and **c** *y*-axis. Reproduced with permission [207]. Copyright 2023, American Chemical Society. **d** The evolution process of design from a scale-integument structure inspired by the snakeskin to a Kiri-MM E-armor. **e** Normalized relative resistance change vs. strain for SECs with Ag contents of 65, 70, and 75 wt.%. Reproduced with permission [211]. Copyright 2022, Wiley‐VCH GmbH. **f** Schematic illustration showing the omnidirectional printing of Ag/MWCNT/PDMS SECs and the emulsion-based ink. **g** Photographs showing the elastic recovery of an SEC after being bent by an external force. **h** Resistance changes of the SECs under strains of 30 and 50% for 3,000 cycles. Reproduced with permission [218]. Copyright 2023, Springer Nature Limited

Compared with its 1D counterpart, the 2D buckling structure shows more complex buckling behaviors, including in-plane wrinkles and irregular wave shapes. Generally, the conductor is designed with geometric features such as wrinkles, ripples, or a fishing net-like configuration to realize the buckling structure. When the conductor is stretched or bent, these wrinkled, corrugated, or reticular geometric configurations allow for deformation to a certain extent without fracturing or losing conductivity as readily as traditional rigid conductors. Such designs impart adequate two-directional stretchability to intrinsically rigid or non-stretchable conductors, enabling them to adapt to a variety of complex deformations and stresses [204–207]. Zhao et al. [207] prepared an SEC with multidirectional stretchability through a pre-stretching process (Fig. 9a–c). Initially, the PDMS base was stretched by 90% bidirectional along the X/Y axis and placed in an ultraviolet/ozone environment to oxidize the surface and enhance its viscosity. Subsequently, single-sided, sticky polyethylene terephthalate tape was attached to the treated PDMS surface, and CuNWs dispersion was sprayed on the back of the tape. After acid etching, a disordered conductive network was formed. This Cu nano-network was then encapsulated with a chitosan layer. Upon release of the substrate pre-strain, the CuNWs/chitosan composite layer spontaneously formed a wavy fold structure. Under 50% tensile strain, the sheet resistance of the fabricated multidirectional SEC remained constant. When the strain increased to 70%, the sheet resistance grew by only 5%, attributed to the buffering effect of the fold structure on the deformation and the anchoring effect of chitosan on the CuNWs. After 1,000 cycles of 70% tensile strain in a 2D direction, the sheet resistance of the multidirectional SEC remained unchanged.

The snakeskin structure is normally composed of a series of parallel wavy or curved lines, which can be made of metal, conductive polymer, or other conductive materials. These lines are arranged at certain intervals in the plane of the conductor, forming a grid-like structure similar to snakeskin scales. When the conductor is stretched or bent, the wavy or curved lines in the snakeskin structure can stretch or bend with the deformation without breaking or losing electrical conductivity [208–212]. For example, Jiang et al. [211] proposed an SEC that not only exhibits mechanical flexibility and electronic functionalities similar to electronic skin but also offers self-protection and protection for underlying software from external physical damage (Fig. 9d, e). The geometry of the mechanical metamaterial (MM) ensures auxetic stretchability and large areal coverage for sufficient protection. Analogous to the composition of snakeskin, the SEC was composed of rigid tiles connected by soft materials at selected nodes within the MM pattern. The MM pattern’s soft hinges were made of a soft polymer composite (silicone rubber K-704 doped with 70 wt% Ag), which endows the SEC with a high conductivity (2.84 × 10^3^ S m^−1^). Material failure, indicated by a sudden increase in resistivity, occurred when stretched to 78% strain.

The mesh structure refers to a grid-like design constructed from small lines or fibers. This structure allows the conductor to move its internal lines or fibers without breaking or losing electrical conductivity when subjected to deformations such as stretching and bending [213–216]. For example, Xu et al. [216] developed a transparent mesh SEC composed of the LM (Galinstan) and PDMS. The SEC was based on a PDMS elastomer, and a PDMS/LM composite ink was filled into the mesh mold using the blade scraping method. After curing, the conductive network was activated by mechanical sintering. The LM was uniformly distributed in the PDMS matrix in the form of microdroplets, and the applied mechanical pressure disrupted the oxide layer and formed a continuous conductive pathway. The design of the network structure provided the SEC with high light transmission (up to 62%) and stretchability (elongation at break > 150%). Zhou et al. [217] prepared a transparent SEC with in-plane stretchability. They employed a breath-figure method to prepare a porous honeycomb pattern as a template for the deposition of an Ag mesh film, and a PVA stamp was then adopted to transfer the Ag mesh film to a PDMS layer. The Ag mesh/PDMS SEC exhibited almost identical Δ*R*/*R*_0_ values in plane of three tensile directions under the same strain within the range of 0–25% and could withstand 800 stretching/releasing cycles under a strain of 20%.

### 3D Stretchable Structure Designs

1D and 2D stretchable structure designs are limited by their uniaxial or biaxial deformation mechanisms, making them challenging to adapt to omnidirectional deformation requirements in complex 3D scenarios. In contrast, 3D stretchable structure designs address these dimensional limitations through unique spatial topological designs, enabling coordinated deformation in 3D space [218]. Lee et al. [218] fabricated 3D-structured SECs through an omnidirectional 3D printing technique based on an emulsion-based composite ink (Fig. 9f–h). They developed a printable ink by dispersing Ag particles and multiwalled carbon nanotubes (MWCNTs) in PDMS, followed by the addition of diethylene glycol (DEG) and chloroform (CHCl_3_). This ink addressed the limitation that traditional inks can only be deposited in a layer-wise manner, enabling the direct printing of 3D structures. The prepared 3D-structured Ag/MWCNT/PDMS SECs exhibited stretchability of up to 160%, and the *R*/*R*_0_ values remained stable within 3.0 and 5.5 under strain of 30 and 50% strain over 3,000 cycles, respectively.

## Summary of This Chapter

The stretchability of SECs can be realized through structural designs, which can be divided into 1D, 2D, and 3D stretchable structures based on the number of stretchable dimensions. 1D stretchable structure designs achieve conductive stability under uniaxial deformation primarily by employing four topological forms: buckling, spiral, wave, and kirigami. The buckling structure adopts a multistage interfacial bending deformation mechanism induced by pretension. The spiral structure utilizes a strain-progressive dissipation strategy via its spring-like geometry. The wavy structure dynamically adjusts the steric hindrance of adjacent elements to diffuse the stress. The kirigami structure releases internal stress by altering its surface shape. 2D stretchable structure designs realize the reconstruction of the conductive network through routes mainly including bidirectional pre-stretching, encompassing buckling configurations, snakeskin bionic metamaterial interconnection designs, and mesh topological deformation compensation mechanisms. The snakeskin bionic structure can realize 2D stretchability through the composite construction of rigid elements and flexible nodes, while the mesh system can maintain 2D electromechanical stability through the topological tunability of the continuous conductive network. 3D stretchable structure designs overcome dimensional limitations and enable coordinated deformation in the 3D space. These structural design paradigms break through the stretchability limitations of traditional materials and provide customizable mechanical adaptation solutions for SECs under complex deformation scenarios. Table 2 summarizes the typical characteristics of SECs with 1D, 2D, and 3D structure designs.

**Table 2:** Typical characteristics of SECs with 1D, 2D, and 3D structure designs

	Structure design	Material	Conductivity or sheet resistance	Stretchability (%)	Δ*R*/*R*_0_ under strain	Preparation Process	Reference
1D	Buckling structure	AgNWs/ZnS:Cu/PDMS	26.8 Ω sq^−1^	180	–	Spray coating	[183]
		AgNPs/PU	26,128 S m^−1^	200	0.005 under 10% strain	Electrochemical deposition	[185]
	Spiral structure	PEDOT@ BC/CNT	–	1175	0 under 100% strain	Wet spinning and coiling process	[189]
		Gallium/PU	–	1273	0.09 under 100% strain	Direct curing method	[190]
	Wave structure	Tetramethylammonium hydroxide/PDMS	–	25	0.02 under 25% strain	Microfabrication process	[195]
		PDMS/MWCNTs	–	40	0 under 40% strain	3D printing process	[193]
	Kirigami structure	Ag/Pd/Cu/PU	–	50	1.5 under 50% strain	Magnetron sputtering	[197]
		EGaIn/Au/silicone rubber	–	820	0.33 under 820% strain	Laser cutting and sputtering	[199]
2D	Buckling structure	Cu NWs/PDMS	–	70	0.05 under 70% strain	Spray coating	[207]
	Snakeskin structure	Ag/silicone rubber	2840 S m^−1^	240	0 under 60% strain	Mechanical cutting	[211]
	Kirigami structure	AgNW/PI	–	50	0.0015 under 50% strain	Laser cutting and sputtering	[212]
	Mesh structure	Galinstan/PDMS	1.2 × 10^4^ S m^−1^	> 150	< 0.09 under 60% strain	Casting molding and mechanical sintering	[196]
3D	3D complex structures	Ag/MWCNTs/PDMS	6682 S cm^−1^	160	–	3D printing process	[218]

## Fabrication Techniques for SECs

Each type of SECs benefits from specific preparation methods, and the selection of fabrication techniques depends on a consideration of customary needs such as desired electrical or mechanical performance, preparation cost, and process feasibility [219–221].

### Fabrication Techniques for Metal-Based SECs

The essence of the fabrication of metal-based SECs involves forming a metal film on the surface of a stretchable substrate, which can be manufactured through methods such as rotary evaporation, magnetron sputtering, and electroless deposition (ELD). Note that since the fabrication techniques for LM-based SECs are mentioned in Sect. 2.1, we will mainly review the fabrication techniques for solid metal-based SECs in this section. The thickness of the solid metal film is generally controlled to be less than tens of nanometers to ensure that the obtained SECs possess both good conductivity and certain stretchability. While these preparation processes are relatively simple and low cost, they still encounter challenges such as nonuniformity, undesirable stability, and limited tensile performance [222–225].

**Fig. 10:**
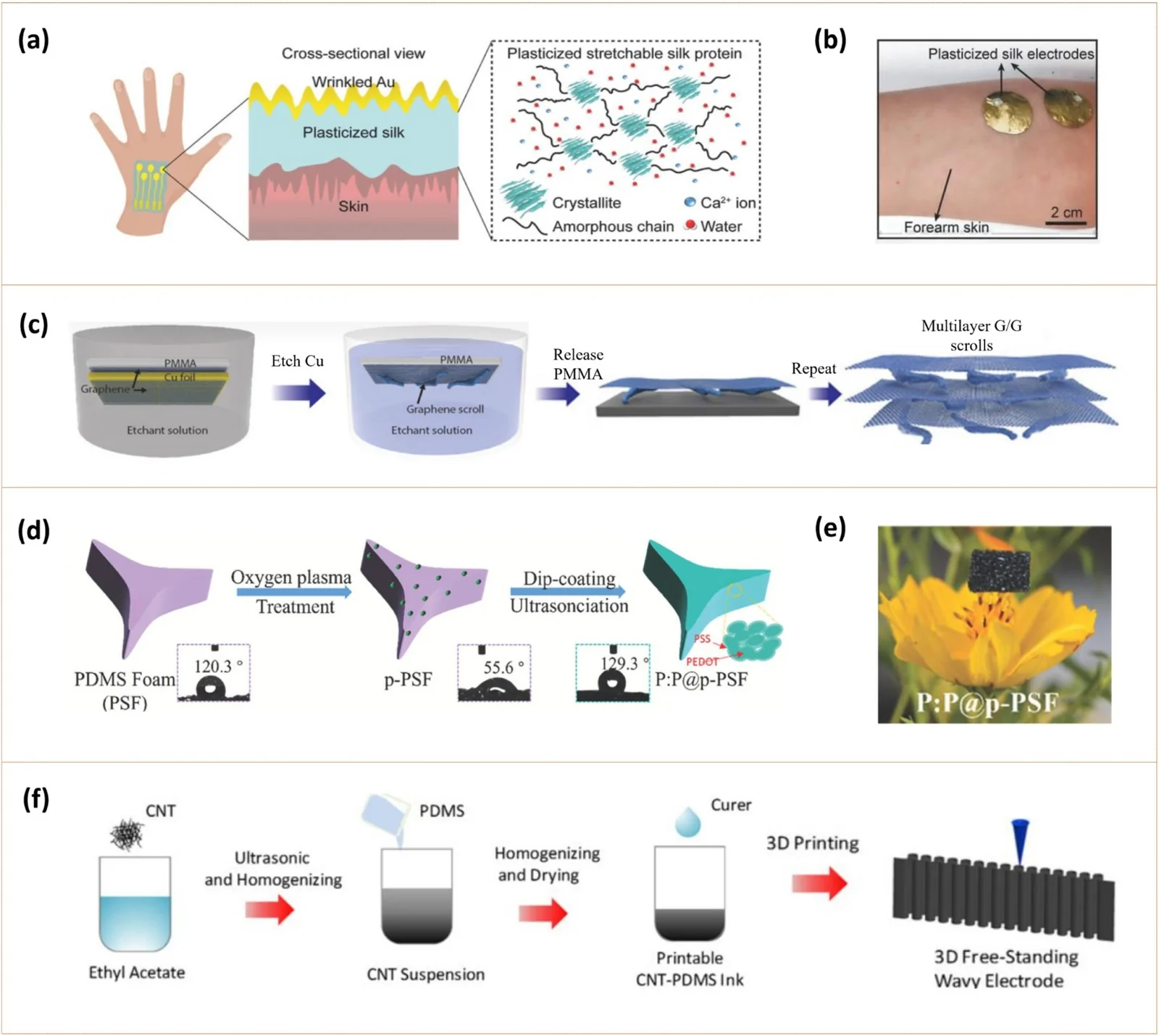
Fabrication techniques for SECs. **a** A scheme of the on-skin SEC based on Au film deposited on the surface of plasticized silk protein by vacuum sputtering. **b** Photograph of an EMG measurement setup using the SECs laminated on a human forearm. Reproduced with permission [230]. Copyright 2018, WILEY‐VCH Verlag GmbH & Co. KGaA, Weinheim. **c** Schematic illustration of the fabrication rocess for MGGs as an SEC. Reproduced with permission [241]. Copyright 2017, American Association for the Advancement of Science. **d** Schematic illustration for the fabrication process of the PEDOT:PSS@PDMS-PSF SEC. **e** Photograph of the PEDOT:PSS film loading on pistils of a flower. Reproduced with permission [251]. Copyright 2024, Wiley‐VCH GmbH. **f** Schematic illustration of the 3D printing processes of SECs with designs of serpentine wavy structures. Reproduced with permission [297]. Copyright 2017, WILEY‐VCH Verlag GmbH & Co. KGaA, Weinheim

The preparation of metal films on stretchable substrates can be broadly categorized into physical and chemical methods. (1) Physical methods primarily encompass evaporation and sputtering. The evaporation method involves heating the metal material to its evaporation temperature so that it vaporizes and then condenses as a thin film on the surface of a substrate. The sputtering method is to bombard a metal target with an ion beam in a vacuum chamber, and the metal atoms on its surface are detached and deposited onto the substrate. Sputtering offers advantages such as low-temperature deposition of high-quality films, large-area deposition on non-single-crystal substrates, strong target selectivity, and good film adhesion [226–230]. Chen et al. [230] took filament protein as the base of an SEC and worked with CaCl_2_ and environmental water to plasticize the protein (Fig. 10a, b). An Au film was deposited onto the protein surface using vacuum sputtering, and a fold structure was formed through ambient hydration. The initially high Young’s modulus (5–12 GPa) and low stretchability (< 20%) of the original filament protein were modified to 0.1–2 MPa and > 400%, respectively, achieving an SEC with high stretchability (> 100%). The initial sheet resistance of the 40 nm Au film on the stretchable filament was 7 Ω sq^−1^, and the *R*/*R*_0_ at 40% strain was 2.45. (2) Chemical methods mainly contain ELD and electroplating. ELD is an autocatalytic redox reaction that enables the deposition of thin metal films on almost all flexible and rigid substrates. The electroplating method leverages metal ions in an electrolyte solution to deposit a metal film onto the surface of a conductive substrate via an electrochemical reaction [231–233]. Zhang et al. [233] developed a surface modification technique to successfully construct a metal (Cu, Ni, Ag) conductive layer with high adhesion by implementing the ELD technology on a PDMS substrate. The adhesion to the substrate was enhanced by polydopamine surface modification. Subsequently, an ethanol-glycol composite ink containing Ag nitrate was spin coated on the modified surface and treated with 1000 mbar atmospheric pressure plasma for 30 min to promote the reduction and fixation of Ag ions. Finally, the Ag-PDMS was immersed into the Cu plating bath, and the Cu-PDMS SEC was achieved through the ELD process. The obtained Cu-PDMS SEC exhibited a conductivity of up to 1.2 × 10^7^ S m^−1^, approaching that of bulk Cu (5.96 × 10^7^ S m^−1^). It maintained stable conductivity under 700% tensile strain, with a resistance change rate of less than 5% after 5,000 cycles of stretching/releasing.

### Fabrication Techniques for Inorganic Nonmetallic Materials-Based SECs

The preparation of inorganic nonmetallic materials-based SECs involves forming an inorganic nonmetallic film on the surface of a stretchable substrate, which can be manufactured through techniques including solution spin coating, vacuum filtration, and layer-by-layer self-assembly. Typical examples are discussed below.

1. Inorganic nonmetallic nanomaterials-based SECs can be fabricated by spin coating a solution/suspension onto an elastomer surface, or filtering the nanomaterials-contained suspension by vacuum to form films and then transferred to elastic substrates [234–237]. For example, Liu et al. [237] developed an SEC based on a folded structure of MXene/single walled CNTs (SWCNTs) double-layer composite film. The process began by mixing the 2D MXene and 1D SWCNTs in an aqueous dispersion. A hybrid conductive network was then formed through vacuum filtration. Finally, the conductive network was transferred to a pre-stretched elastic substrate (3 M VHB tape) and released. An SEC with a folded structure was thus obtained. Its conductivity could reach 3.01 × 10^3^ S m^−1^, and Δ*R*/*R*_0_ was about 0.38 at 500% tensile strain, indicating high conductive stability.
2. Inorganic nonmetallic nanomaterials-based SECs can be fabricated via processing into multi-layer stacked structures using layer-by-layer self-assembly or decal transfer methods. Layer-by-layer self-assembly is generally achieved by alternately immersing the substrate in a nanosheet dispersion solution with opposite charges (e.g., negatively charged MXene and positively charged rGO), using electrostatic adsorption to achieve a step-by-step stacking of layered nanomaterials. The decal transfer method involves synthesizing a layer of inorganic nonmetallic nanomaterials on a rigid substrate (e.g., graphene grown on a Cu foil via CVD), followed by the spin coating of a polymeric sacrificial layer (e.g., polymethyl methacrylate (PMMA)). Subsequently, the rigid substrate is etched away through chemical processing, and the acquired film is transferred as a whole to the target stretchable substrate [238–241]. For example, Liu et al. [241] developed a transparent, conductive graphene structure called a multilayer graphene/graphene vortex (MGG), achieved by inserting graphene scrolls between graphene layers (Fig. 10c). Initially, graphene was grown on a Cu foil through a CVD method, with the foil suspended in the center of a CVD quartz tube to allow graphene growth on both sides. The obtained G/Cu/G structure was then spun onto a thin layer of PMMA to protect one side of the graphene. Afterward, (NH_4_)_2_S_2_O_8_ was utilized to etch away the Cu foil in the entire film. The bottom graphene, without PMMA coating, formed a graphene scroll due to surface tension. The MGG structure was obtained by repeating this transfer process multiple times on the same substrate. The MGG structure could retain 65% of the original conductivity at 100% strain perpendicular to the current flow direction and 60% of its original current output at 120% strain parallel to the charge transfer direction.

### Fabrication Techniques for Conductive Polymer-based SECs

The preparation of conductive polymer-based SECs involves a multi-scale collaborative regulatory strategy to balance the inherent conflict between the material’s electrical conductivity and mechanical stretchability. Three primary fabrication techniques are commonly employed: solution treatment, in situ polymerization, and laser-induced technique [242–249].

1. The solution treatment technique is mainly to deposit a conductive polymer solution on an elastic substrate by means like spraying, spin coating, and immersion, or to fabricate accurate conductive patterns exploiting a conductive polymer solution/ink by means like inkjet printing and 3D printing [250–254]. For example, Nie et al. [252] proposed a PEDOT:PSS/PDMS SEC (Fig. 10d, e). As a first step, the PDMS was treated by a foaming process to form a porous elastic foam (PSF). The PSF was then treated with O_2_ plasma to activate its surface. Finally, the activated PSF was immersed in a PEDOT:PSS aqueous solution, with ultrasonic assistance to facilitate infiltration of PEDOT:PSS into the PSF, followed by drying. The resulting PEDOT:PSS/PDMS SEC exhibited a Δ*R*/*R*_0_ value of about 97.4% at 60% compression strain.
2. The in situ polymerization technique involves triggering the chemical or electrochemical polymerization of conductive polymers directly on the elastic substrate surface (e.g., PEDOT grown on PDMS via oxidative CVD) to enhance interfacial bonding [255–257]. For example, Li et al. [257] proposed an SEC with a pleated PPy coating on PU (PU@PPy). The PU fibers were pre-treated by soaking in an ethanol solution containing pyrrole for pre-treatment and then immersed in FeCl_3_·6H_2_O and sodium sulfonyl salicylate (NaSSA) composite solution for in situ polymerization at a low temperature of 2 °C. This process resulted in a uniform conductive coating of PPy. The doping with NaSSA could improve both the electrical conductivity (to 634 S m^−1^) and the stretchability (to a fracture strain of > 100%) of PPy. The obtained SEC exhibited an initial conductivity of 634 S m^−1^, Δ*R*/*R*_0_ of 3.5% under 50% tensile strain, and an elongation at break of approximately 850%.
3. The laser-induced technique is capable of directly bonding PEDOT:PSS to various polymer substrates through photothermal reaction in the interface induced by a laser, which enables the patterning of PEDOT:PSS on polymer substrates with micrometer-scale resolution [249, 258]. Won et al. [258] developed a PEDOT:PSS hydrogel-based SEC by a laser-induced process, which stably adheres patterned pure PEDOT:PSS hydrogel to polymer substrates through a continuous-wave 532 nm laser-induced phase separation and interface structures. After the laser scanning process, the PEDOT:PSS was dipped in ethylene glycol to strengthen the interconnections between the PEDOT-rich domains. The PEDOT:PSS hydrogel-based SEC possessed a wet electrical conductivity of up to 101.4 S cm^−1^, peel strength of 64.4 N m^−1^, and lap shear strength of 62.1 kPa.

### Fabrication Techniques for Composite Materials-based SECs

Composite materials-based SECs comprise conductive fillers dispersed in a single or composite polymer matrix. The primary manufacturing objective is to construct a stable and efficient conductive network within a stretchable polymer matrix. Techniques such as solution mixing and molding, electrospinning, screen printing, and 3D printing have been commonly utilized in the preparation of composite materials-based SECs [259, 260].

1. The most common preparation technique for composite materials-based SECs is solution mixing and molding, where conductive fillers (e.g., CNTs, MXene, PEDOT:PSS) [261–264] are dispersed in a liquid elastomer precursor, followed by casting and curing [18, 265–270]. The key to solution mixing and molding is to ensure a uniform dispersion of conductive packing in the polymer matrix, and methods such as continuous stirring, sufficient grinding, and dispersant addition are widely applied to achieve the uniform dispersion.
  Continuous stirring is the most commonly adopted method to achieve even dispersion of conductive fillers [271–274]. Sharma et al. [271] fabricated an SEC using PEDOT:PSS, polyvinylpyrrolidone (PVP), and CNF as raw materials. In the first stage, PEDOT:PSS and PVP were mixed and continuously stirred until they were completely dispersed. Subsequently, CNF was immersed in the solution to obtain PEDOT:PSS-PVP/CNF composites, which were then infiltrated into PDMS for encapsulation. The obtained SEC could withstand repeated bending, folding, and twisting and could recover its original state after the removal of external forces. Its conductivity reached up to 1.08 S cm^−1^. Luo et al. [274] added p-tert-octylphenol (Triton X-100) to PEDOT:PSS to create a mixed solution, which was then mixed with PDMS and stirred vigorously. The uniformly dispersed mixture was placed onto a mold and dried to obtain an SEC that exhibited a minimum sheet resistance of 20 Ω sq^−1^ and elongation at a break of about 82%.
  Sufficient grinding can effectively disaggregate the conductive packing and promote its uniform dispersion in solution [275–278]. Ahn et al. [275] fabricated a snake-like CNT-nanocomposite-based SEC. Initially, CNTs were ground with carboxymethyl cellulose (CMC) in distilled water for 30 min, and the slurry was then squeezed into a PDMS mold for drying to obtain an SEC. The measured minimum resistance of the SEC was 138 Ω, and the elongation at break was around 357%. Xu et al. [278] reported an SEC by mixing a PDMS-based bottle brush elastomer (BBE) with SWCNTs and then solidifying the mixture to form a SWCNT percolation network in the elastomer matrix. The high aspect ratio of SWCNTs (length/width is about 2,500) resulted in good electrical conductivity (> 2 S m^−1^) of the SEC, and a relatively low loading concentration yielded good tensile properties (stretchability > 100%).
  The addition of a dispersant can also promote the uniform dispersion of the conductive filler in the solution [279–281]. Chen et al. [281] constructed an SEC of polyacrylamide (PAAM)-graphene-PANI ternary composite with a multistage conductive pathway through component design and decentralized regulation. The rGO and PANI nanofibers were dispersed in an acrylamide (AAM) monomer solution. On this basis, PVP and lignin were introduced as dispersants to achieve even filler distribution through the steric hindrance effect. Finally, a 3D interpenetrating network structure was formed after polymerization and curing by ultraviolet light. Graphene provides an effective conductive network in the SEC and enhances the electrical stability under tensile strain. At 200% strain, the resistance increased by 5.6 times with graphene, whereas it increased by 16.9 times without graphene. In addition, the tensile strength of the SEC reached 44.31 kPa, and the elongation at break reached 306.7%, higher than that of the SEC without graphene.
2. Electrospinning exploits a high-voltage electric field to stretch a conductive polymer solution into a network of microfibers, forming an SEC with both high flexibility and good conductivity [282–284]. For example, Yin et al. [284] prepared a composite materials-based SEC by uniformly blending rGO and PEDOT:PSS into a PVA solution and then employing the electrospinning technique combined with a high-speed turntable receiving screen to directly regulate fiber arrangement. The acquired SEC exhibited an electrical conductivity of 1.7 S m^−1^ and elongation at a break of 61.13%.
3. Screen printing for the fabrication of SECs involves achieving microscale patterns by combining a conductive paste of high viscosity (e.g., AgNWs/PDMS composite ink) with an elastic substrate (e.g., PDMS, TPU) and a structural design (e.g., island bridge structure), which enables the preparation of SECs over a large area [285–287]. Shang et al. [287] prepared an SEC via screen printing and water jet sintering. Firstly, EGaIn was dispersed in propylene glycol and PVP as the ink, and then, the ink was screen printed on a TPU substrate and water jet sintered. Finally, the SEC was obtained by TPU encapsulation. Its electrical conductivity was as high as 7.3 × 10^5^ S m^−1^. Its resistance increased by only 10% after 500 cycles of 50% strain, and it maintained conductive stability even when stretched to 800%.
4. 3D printing has been employed to build SECs with complex 3D structures. While electrospinning and screen printing can also utilize inks to prepare composite materials-based SECs, 3D printing offers unique advantages [288, 289]. First of all, it enables customized structure designs to meet the needs of SECs for different application scenarios. Secondly, 3D printing technique can manufacture SECs with complex shapes and internal structures with high stability. Thirdly, 3D printing boasts high production efficiency and material utilization [290–293]. The preparation process of SECs by 3D printing technology typically starts with the mixing of the selected polymer matrix and conductive fillers, followed by the addition of solvents and surfactants to formulate 3D printing inks. The prepared ink is generally required to exhibit rapid curing, good interface bonding, and stretchability. It is then printed layer by layer into an SEC with a 3D printer [35, 294–297]. For example, Hong et al. [297] applied 3D printing to manufacture a free-standing SEC (Fig. 10f). Initially, CNTs were dispersed in ethyl acetate, followed by ultrasonic homogenization. PDMS base was then added into the uniform CNTs suspension and homogenized again. Subsequently, the ethyl acetate was evaporated at 80 °C to obtain the PDMS-CNT composite ink. An SEC with a snake-shaped cross section was then fabricated by a 3D printer. The SEC achieved high tensile properties and high electrical stability, with an elongation of 315% at the break at a 45° connection angle and a relative resistivity change of 5% at 100% strain.

## Summary of This Chapter

Different kinds of SECs necessitate distinct preparation approaches. The fabrication techniques for solid metal-based SECs can be mainly divided into physical methods, mainly evaporation and sputtering, and chemical methods, primarily ELD and electroplating. While sputtering allows for low-temperature film formation, it can suffer from the issue of nonuniformity. ELD enables the deposition of thin metal films on almost all flexible and rigid substrates. The preparation of inorganic nonmetallic materials-based SECs can be mainly achieved by techniques including solution spin coating onto an elastomer, vacuum filtration followed by a transfer to elastic substrates, and processing into multi-layer stacked structures with supporting elastic substrates via layer-by-layer self-assembly or decal transfer. Conductive polymer-based SECs are mainly fabricated by the solution treatment, in situ polymerization, and laser-induced techniques. The solution treatment technique employs methods like spraying, spin coating, and immersion to deposit a conductive polymer solution on an elastic substrate; or constructs accurate conductive patterns utilizing a conductive polymer solution/ink by means like inkjet printing and 3D printing. The in situ polymerization technique chemically synthesizes a conductive layer directly on an elastic substrate’s surface. The laser-induced technique can adhere PEDOT:PSS onto a variety of polymer substrates through interface photothermal reaction by using a laser. The most common preparation technique for composite materials-based SECs is solution mixing and molding, which can employ methods like continuous stirring, sufficient grinding, and dispersant addition to ensure uniform dispersion of conductive fillers. In addition, techniques such as electrospinning, screen printing, and 3D printing can be adopted to achieve various patterns/structures of SECs. Specifically, 3D printing overcomes the limitations of traditional manufacturing dimensions through customized ink formulations and layered stacking strategies, enabling the efficient molding of customizable complex 3D structures. Table 3 summarizes the typical fabrication techniques employed for SECs.

**Table 3:** Typical fabrication techniques employed for SECs

Type of SECs	Fabrication technique	Procedures	Typical materials	Advantage	Disadvantage
Metal-based SECs	Rotary evaporation	Metal deposition via thermal evaporation under vacuum	Au, Ag, Cu	Simple process, low cost	Poor adhesion, limited flexibility, uneven thickness
	Magnetron sputtering	High-energy plasma deposition onto substrates	Au, Ag, Cu	High uniformity, good adhesion	Expensive equipment, limited scalability
	Electrochemical deposition	Electrodeposition of metals onto patterned substrates	Cu, Ni, Ag	Low cost, scalable for large areas	Uneven thickness, limited mechanical stretchability
	Microchannel injection	LM infusion into microfluidic channels	EGaln	Complex shape fabrication, high resolution	Complex operation, high cost
	Pressure printing	Pressure-induced LM patterning on elastomer surfaces	EGaln	Low cost, simple process	Uneven thickness, poor adhesion
	Self-assembly of modified LMs	LM self-forming film via laser induction, thermal evaporation, or solvent treatment	EGaln	Controllable cost	Poor uniformity for thick layers, insufficient stability
Inorganic nonmetallic materials-based SECs	Solution coating	Spin/dip coating of conductive solutions	Graphene, CNTs, MXenes	Simple process, low cost	Poor uniformity for thick layers, low durability
	Vacuum filtration	Pressure differential-driven solid–liquid separation	Graphene, CNTs, MXenes	Simple process, low cost, high film uniformity, controllable thickness	Poor mechanical properties, post-processing required
	Layer-by-layer self-assembly	Layer-by-layer deposition of conductive inks	Carbon-based inks, MXenes	Customizable geometries, multifunctional designs	Limited resolution, high ink viscosity requirements
	Decal transfer	Polymer-assisted damage-free transfer of inorganic nonmetallic layers	Graphene, CNTs, MXenes	Lossless transfer, large area transfer	Complex operation, high cost
Conductive polymer-based SECs	Solution treatment	Spin/dip coating of conductive solutions	PEDOT:PSS, PPy	High transparency, solution-processable	Uneven thickness, low durability
	In situ polymerization	Initiating a polymerization reaction on the substrate surface	PEDOT:PSS, PPy, PANI	Homogeneous dispersion	Aggregation issues, high cost
	Laser-induced formation	Adhere conductive polymer on substrates through interface photothermal reaction by using a laser	PEDOT:PSS	Enable formation of complex structures, high resolution	High cost, limited scalability
Composite materials-based SECs	Electrospinning	High-voltage electrospinning of polymer solutions/melts into micro-/nanofibers	rGO/PEDOT:PSS/PVA	High stretchability, large-scale production	Lack of mechanical durability, sensitive process parameters
	Screen printing	Direct micrometer patterning via template scraping of high-viscosity conductive paste	AgNWs/PDMS, EGaIn/TPU	High production efficiency, low cost	Resolution limitations, material waste
	3D printing	Extrusion-based printing of conductive polymer blends	Graphene/PDMS, AgNW/Ecoflex	Complex shape fabrication, on-demand design	Post-processing required, limited material choices

## Applications of SECs

SECs have been adopted as fundamental and crucial components in stretchable electronics, including serving as the electrodes of stretchable devices, functioning as the sensing material components of stretchable sensors, or acting as interconnecting components bridging devices of electronic systems, which are subject to different requirements according to different application scenarios.

### Stretchable Energy Conversion Devices

Stretchable energy conversion devices can maintain their energy conversion functionalities even when subjected to mechanical deformations, such as stretching, bending, and twisting. SECs with high conductivity can help reduce energy loss and improve overall energy conversion efficiency, and SECs with high mechanical stability and durability can help save maintenance costs [298, 299]. Up to now, SECs have been applied in various kinds of energy conversion devices, including nanogenerators, solar cells, and fuel cells.

#### Stretchable Nanogenerators

In stretchable nanogenerators, such as stretchable piezoelectric nanogenerators (PENGs), triboelectric nanogenerators (TENGs), pyroelectric nanogenerators, and thermoelectric nanogenerators, SECs have been employed as the electrodes and functional material components [300–310]. The specific characteristics required for the SECs of these nanogenerators vary depending on the distinct working mechanisms and device structures.

**Fig. 11:**
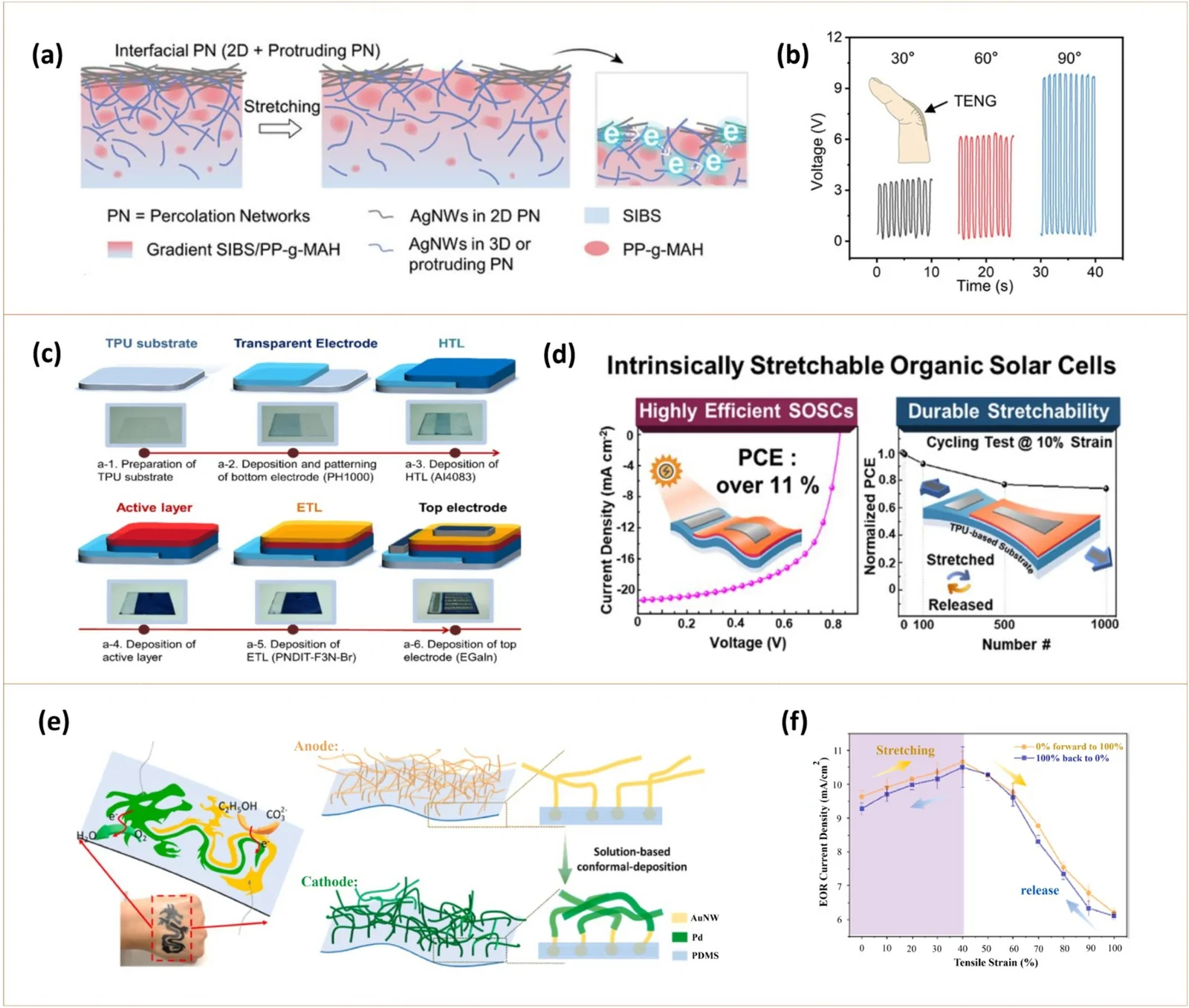
Applications in stretchable energy conversion devices. **a** Schematic diagram of the conduction mechanism of the SEC with interfacial PN under stretching. **b** Output voltage of the TENG with the SEC as the conductive electrode layer for monitoring finger bending at 30°, 60°, and 90°. Reproduced with permission [321]. Copyright 2024, Wiley‐VCH GmbH. **c** Schematic illustration of the fabrication processes for the SOSC with the PEDOT:PSS SEC as the bottom electrode. **d** The photovoltaic performance and durable stretchability of the SOSC with the PEDOT:PSS SEC and EGaIn SEC as the two electrodes. Reproduced with permission [340]. Copyright 2021, American Chemical Society. **e** The design of the dragon-tattoo like epidermal fuel cells with EP-AuNW SEC and EP-AuPdNW SEC as the electrodes. **f** Oxidation peak current densities of the EP-AuPdNW SEC in a stretching/releasing loop within 0–100% strain. Reproduced with permission [355]. Copyright 2022, Elsevier B.V

As for stretchable TENGs and PENGs, which exhibit high internal impedances, especially TENGs, the resistances of their stretchable electrodes can fluctuate within a wide range without affecting their electrical outputs [311–314]. (1) PENGs: Due to the piezoelectric effect, PENGs must undergo mechanical deformation during energy harvesting, which requires the applied SECs which serve as their electrodes or piezoelectric materials to withstand prolonged cyclic strain [315, 316]. Xu et al. [316] prepared a droplet-like porous barium zirconate titanate ceramic using a freeze-casting method and sputtered Au on its surface. Subsequently, the LM (EGaIn) was printed on the polymer substrate according to a specific pattern via 3D printing, yielding a stretchable PENG. The LM electrode helped the device maintain high working stability. After 5,000 stretching/releasing cycles at 60% strain and 5,000 twisting cycles at 180°, the open-circuit voltage of the stretchable PENG remained stable. (2) TENGs: When an SEC functions not only as an electrode of a TENG but also as one of the two triboelectric layers, it is advantageous to have a significant difference in its tendency to gain or lose electrons from the other triboelectric layer [317–320]. This disparity facilitates the generation of more triboelectric charges upon contact between the two triboelectric layers, contributing to higher electrical outputs. Specifically, in a sliding-mode TENG, where triboelectric layers are more prone to wear, the SEC as one triboelectric layer also needs to possess strong abrasion resistance. Zhang et al. [321] developed an SEC with high performance through constructing an interfacial percolation network (PN), which integrates a 2D AgNWs PN and a protruding 3D AgNWs PN (Fig. 11a, b). The protruding PN was formed by introducing polypropylene-graft-maleic anhydride domains in the near-surface region of a poly(styrene-isobutylene-styrene) (SIBS) elastomer matrix, causing AgNWs to change from horizontal to quasi-vertical arrangement and protrude out. The SEC achieved a conductivity of 13,500 S cm^−1^ under static conditions and elongation at a break of 660% strain. This SEC was applied as the conductive electrode layer in a single-electrode-mode TENG. The TENG showed an output voltage of about 60 V under 300% strain and could effectively monitor finger bending at 30°, 60°, and 90° by generating distinct voltage signals.

As for stretchable pyroelectric and thermoelectric nanogenerators, which operate in environments with temperature fluctuations or temperature gradients [322, 323], the SECs applied as their electrodes need to possess high thermal stability [324–327]. It has been reported that SECs (e.g., LM-based SECs) replacing traditional rigid conductive materials as the interconnects and thermal interface materials can help improve the performance involving enhancing the interface thermal conductivities and mechanical stability [328]. Additionally, SECs owning thermoelectric effect can serve as thermoelectric materials in thermoelectric nanogenerators, which are favored to possess a high Seebeck coefficient, high electrical conductivity, and low thermal conductivity in order to have high thermoelectric conversion efficiency [329, 330]. He et al. [329] developed a stretchable CNT/PVP/PU conductive textile via sequential electrospinning and air pressure spraying processes to serve as a core component of wearable thermoelectric devices. The fabrication involved first electrospinning PU nanofibers to create a breathable substrate, followed by the spray coating of CNT dispersions stabilized with PVP. The PU acted as the supporting skeleton, the CNT served as the thermoelectric material and the PVP not only improved the dispersion of CNTs but also served as interfacial binders between the CNT and PU. This hierarchical architecture, combining elastic polymer skeleton with conductive CNT networks, yielded 250% elongation and 425 mm s^−1^ air permeability. By serially connecting five layers of the optimized CNT/PVP/PU conductive textile into a thermoelectric device, they achieved room-temperature voltage generation of 0.75 mV through harvesting body heat. Chai et al. [324] developed a (4-aminotetrahydropyran)_2_PbBr_2_Cl_2_ (APBC)-polycarbonate (PC)@poly(vinylidene fluoride-trifluoroethylene) [P(VDF-TrFE)] core sheath nanofiber SEC via coaxial electrospinning as the electrode in a pyroelectric device. The fabrication process of this SEC involved dissolving organic–inorganic perovskite APBC crystals into a PC core solution, while P(VDF-TrFE) served as the sheath material of the SEC. Through precise electrospinning control, flexible fibers having diameters of 300–700 nm were formed in the SEC, with APBC uniformly embedded in the PC core and P(VDF-TrFE) encapsulating the periphery of the SEC. This hierarchical structure exhibited a pyroelectricity of 58.2 μC m^−2^ K^−1^ at 333 K, where the pyroelectric effect originated from spontaneous polarization changes under temperature fluctuations.

#### Stretchable Solar Cells

Stretchable solar cells, which convert solar light into electricity [331–333], are typically composed of a photoactive layer, transparent electrode, back electrode, protective layer, interconnecting component, and encapsulation layer [334–336]. SECs have been applied as the electrodes, interconnecting components, and photoactive layers in stretchable solar cells. When employed as the transparent electrode, the SEC is required to exhibit both high transparency to maximize light transmission to the light absorption layer and high conductivity to ensure efficient electron transport. When employed as the back electrode, the SEC is required to have both high conductivity and high reflectivity to trap light inside the device [337–340]. Noh et al. [340] integrated a TPU substrate, PEDOT:PSS and EGaIn electrodes to fabricate stretchable organic solar cells (SOSCs) (Fig. 11c, d). Among them, PEDOT:PSS modified with polyethylene glycol and citric acid was utilized as the transparent electrode in SOSCs. A precise spray coating system was utilized for atomizing the EGaIn electrode. The SOSCs retained over 74% of their original performance even after 1,000 cycles at 10% tensile strain.

When employed as an interconnecting component in solar cells, the SEC connects different functional layers and is preferred to possess sufficient stretchability and mechanical strength [341]. Liu et al. [341] embedded electrospun polypropylene fibers in Ecoflex as an elastic substrate and coated its surface with a semi-LM (EGaIn doped with Ag-coated Cu particles) to prepare SECs. These SECs acted as an interconnecting component for solar cell arrays, connecting rigid monocrystalline silicon solar cells to maintain a stable electrical connection when stretched, bent, and twisted. The SEC delivered a conductivity of as high as 6 × 10^6^ S m^−1^ and exhibited excellent mechanical properties, with a single structural unit achieving elongation at a break of 200% and remaining stable after 5,000 stretching/releasing cycles. The short-circuit current of the whole solar cell array decreased by only 0.22% under 100% tensile strain.

When the SEC serves as a photoactive layer in a solar cell, it is responsible for converting solar energy into electricity, so it requires not only high optical absorption and charge transport capabilities but also mechanical stability, chemical durability, and good compatibility with other functional layers [342, 343]. Lee et al. [343] obtained a block copolymer PDs (D18_0.8_-s-PEHDT_0.2_) through the chemical bonding of rigid poly[(2,6-(4,8-bis(5-(2-ethylhexyl-3-fluoro)thiophen-2-yl)-benzo[1,2-b:4,5-b′]dithiophene))-alt-5,5′-(5,8-bis(4-(2-butyloctyl)thiophen-2-yl)dithieno[3′,2′:3,4;2″,3″:5,6]benzo[1,2-c][1,2,5]thiadiazole)] (D18) and flexible poly[bis(2-hexyldecyl) 5-(4,8-bis(5-(2-ethylhexyl)-4-fluorothiophen-2-yl)-6-methylbenzo[1,2-b:4,5-b′]dithiophen-2-yl)-5″-methyl-[2,2′:5′,2″-terthiophene]-3,3″-dicarboxylate] (PEHDT). This stretchable copolymer acted as the photoactive layer in the fabricated solar cell. The D18 block maintained excellent light absorption and charge transport properties, while the PEHDT block maintained exceptional tensile properties. The SOSCs achieved a power conversion efficiency of 14.3% while retaining 80% of their initial efficiency at 31% strain.

#### Stretchable Fuel Cells

Stretchable fuel cells convert chemical energy into electrical energy, in which SECs have been mainly applied as the electrodes, current collectors, and interconnecting components [344–347]. The SEC as the stretchable electrode is typically composed of a conductive and electrochemically active material integrated with a stretchable polymer matrix. It acts as the primary site for electrochemical reactions and is responsible for receiving and transmitting electrons. Its stretchability can be achieved through specialized structural designs (wavy, serpentine, etc.) or intrinsic stretchability [348–350]. The SEC as the stretchable current collector is typically a highly conductive material, such as a metal mesh and conductive polymer, supported by a stretchable polymer substrate, which is mainly responsible for collecting the current generated on the electrode and transmitting it to the external circuit [351–353]. Interconnecting components are usually made of stretchable conductive materials that connect electrodes with current collectors to form a complete circuit for electron transmission, and ensure the smooth progress of internal chemical reactions in the fuel cell during the connection process, such as providing suitable transmission paths for reactive gases or liquids [17, 354].

The SEC is essential for the performance stability of stretchable fuel cells in practical applications [355]. Lu et al. [355] presented a tattoo-like epidermal fuel cell based on Pd conformally-coated and one-end-embedded percolation Au nanowire (EP-AuNW/EP-AuPdNW) networks (Fig. 11e, f). Among them, EP-AuNW and EP-AuPdNW, combined with PDMS, were applied as the electrodes in the stretchable fuel cell, with EP-AuNW acting as the anode, and EP-AuPdNW as the cathode. The ultra-long Au NWs, unable to stand vertically, lay on the elastic surface to form a stacked permeable conductive network. Pd adhered to the surface of AuNW to form a uniform and stable film, which enhanced the completeness of the conductive pathway, ensuring conductivity during stretching. The EP-AuPdNW electrode maintained its initial electrocatalytic performance under 60% strain. The fuel cell could operate under a variety of mechanical deformations, including tension, compression, bending, and torsion, retaining 75% of its performance even at 80% strain.

Specially designed structures for the SEC in a stretchable fuel cell can help improve the device’s performance [356]. Zhai et al. [356] proposed a stretchable fuel cell with flammulina velutipes-like vertically aligned Au NWs (v-AuNWs) embedded into a fully cured PDMS film as the stretchable electrodes. The current density of the fuel cell with the tail-exposed (the growing end in contact with the base) v-AuNWs electrode was higher than that of the fuel cell with the head-exposed (the upward end when growing) v-AuNWs electrode. The tail-exposed v-AuNWs electrode served as the anode, and the Pt-modified tail-exposed v-AuNW acted as the cathode. The fuel cell with these stretchable electrodes exhibited high overall performance, with a power density of 80 μW cm^−2^, a current density of 0.475 mA cm^−2^, and a stretchability of 50% tensile strain. Even at 50% strain, the power density of the fuel cell was 47 μW cm^−2^, approximately 60% of its initial power density.

### Stretchable Energy Storage Devices

Stretchable energy storage devices, generally referring to stretchable batteries and supercapacitors, provide stable power for stretchable electronics, and SECs are an indispensable component to maintain their normal functions [357–362].

#### Stretchable Batteries

The storage of energy in batteries normally involves the insertion and extraction of ions into electrodes [363–366], and SECs have been employed as both the current collectors and electrodes of stretchable batteries. SECs as the stretchable current collectors are primarily responsible for collecting the current generated at the electrode and transporting it to the external circuit, which need to meet the requirements of high conductivity, high electrochemical stability, firm combination with electrochemically active electrodes, and good tensile properties. The preparation process and properties of stretchable current collectors directly affect the overall performance of stretchable batteries [367–373]. Gu et al. [373] developed an SEC based on gradient-assembled AuNPs/PU to serve as the current collector in stretchable lithium-ion batteries. The SEC was assembled by vacuum-assisted filtration to form a gradient multilayer structure (90/50/90 wt%). The outermost layer was a highly conductive film composed of 90 wt% AuNPs and WPU, while the middle layer was a lower-content compound of 50 wt% AuNPs. The interface avoided stratification through strong interaction. The stretchable battery with the SEC as the current collector provided a specific capacity of 100 mAh g^−1^ at a current density of 0.5 A g^−1^ and a capacity retention rate of 96% after 1,000 cycles of charging/discharging at a current density of 0.5 A g^−1^.

**Fig. 12:**
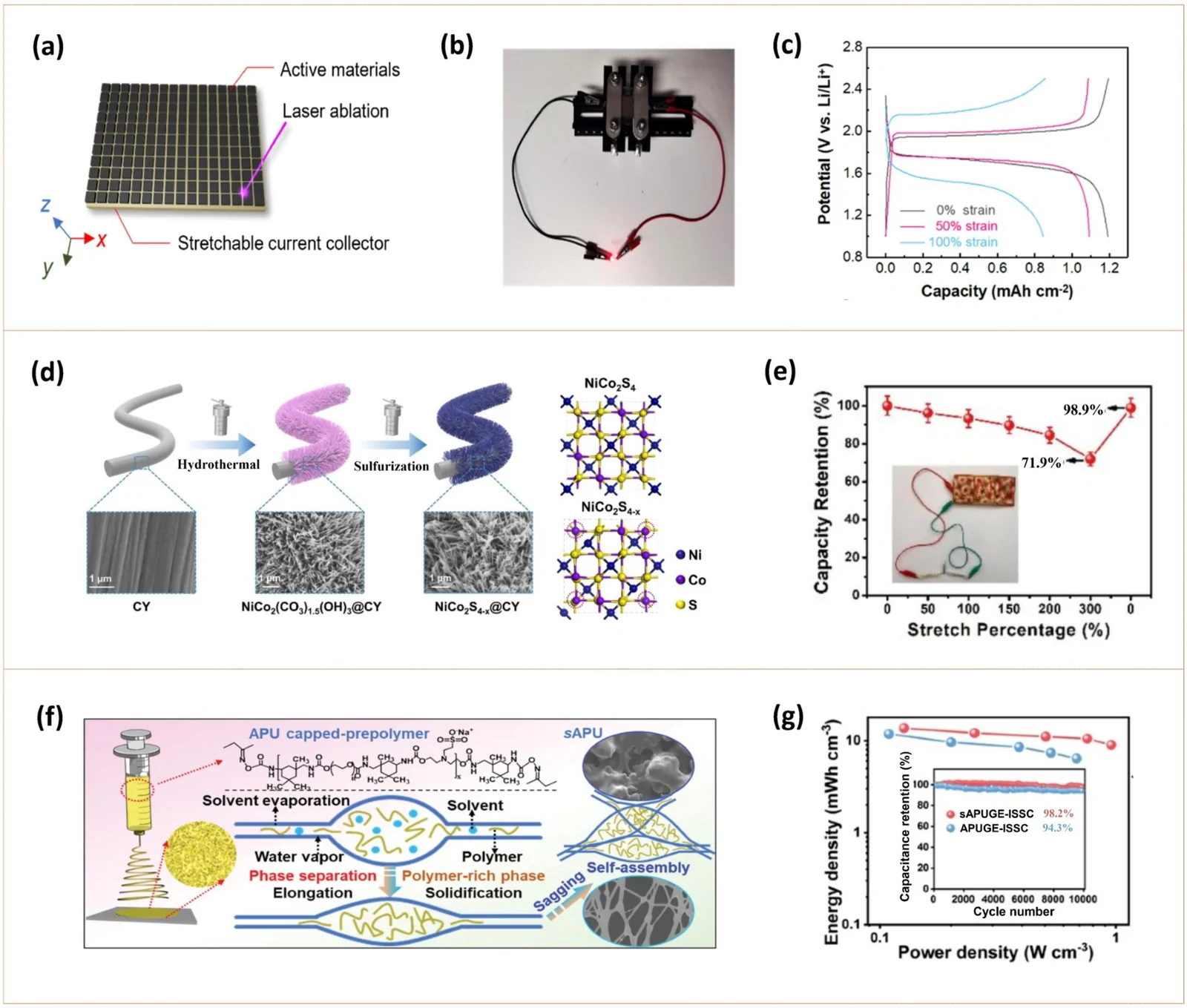
Applications in stretchable energy storage devices. **a** Schematic diagram of the microarray SEC fabricated by laser ablation. **b** Photograph of a light-emitting diode bulb lighted by the stretchable battery with the SECs as the electrodes. **c** Discharging/charging voltage profiles of the stretchable battery with the SECs as the electrodes in the unstretched, 50% stretched, and 100% stretched states. Reproduced with permission [377]. Copyright 2022, Elsevier Inc. **d** Schematic diagram showing the fabrication process of the NiCo_2_S_4_ − x@CY SEC. **e** The capacity retention of the yarn-based zinc-ion battery with the SEC as the electrode under strain from 0 to 300%. Reproduced with permission [378]. Copyright 2023, Donghua University, Shanghai, China. **f** Schematic diagram of the preparation process and fabrication mechanism for sAPU. **g** The Ragone plots of the as-assembled sAPUGE-ISSC and APUGE-ISSC with carbon-based SECs as the electrodes. The inset is the cycling performance at a current density of 5 mA cm^−2^. Reproduced with permission [392]. Copyright 2022, Wiley‐VCH GmbH

The SECs as the stretchable electrodes not only need to fulfill the functions of traditional battery electrodes but also are required to maintain stable electrochemical properties under mechanical deformations such as stretching, bending, and twisting. They serve three main functions in stretchable batteries: (1) electron conduction and charge transfer; (2) active material loading and interface reaction; and (3) mechanical support. Special electrode structures, such as a patterned electrode structure, can be designed to improve the electrochemical performance of the stretchable battery. These unique structures can ensure stable contact and ion transport between key components of electrodes and electrolytes when the battery is stretched or bent, thereby preserving the key performance of the battery [374–378]. For example, Lu et al. [377] proposed SECs fabricated by laser ablation of active material films and employed them as the electrodes of stretchable lithium-ion batteries (Fig. 12a–c). The Li_4_Ti_5_O_12_ or LiFePO_4_ active material was mixed into a paste with carbon black and PVDF in N-methyl-2-pyrrolidone. The obtained viscous slurry was coated on an Ag–Cu/Ni carbon-based conductive silicone substrate. Subsequently, the SEC was formed with an independent micrometer square array structure by laser ablation. The Li_4_Ti_5_O_12_-based SEC acted as the anode and the LiFePO_4_-based SEC served as the cathode. The structure alleviated stress concentration through micro-discretization of the active material. The microarray design allowed the conductors to maintain conductive network continuity under stretching, while a high load capacity of 10 mg cm^−2^ ensured energy storage capacity. Combined with a gel electrolyte and PDMS packaging, the stretchable battery retained 90.2 and 70.9% of its capacity under 50 and 100% strain, respectively, with a surface capacity of 1.2 mAh cm^−2^. After 500 cycles of stretching/releasing at 100% strain, the SECs as the electrodes exhibited a slight decrease in specific capacity, with approximately 5% degradation observed, showing good strain adaptability and stability. Cheng et al. [378] developed an SEC of NiCo_2_S_4-x_@carbon yarn (CY) composite as the battery electrode based on a sulfur vacancy regulation strategy (Fig. 12d, e). The SEC was constructed as a hollow nanotube array on the surface of the conductive CY via a two-step hydrothermal process. Specifically, sulfur vacancies (local defects formed by ion exchange blocking) were induced by adjusting the concentration of sulfur precursors, and hollow nanotube structures were then formed by the Kirkendall effect. The electron delocalization effect induced by the sulfur position optimizes the charge transfer path, reducing the charge transfer resistance to 1.314 Ω and achieving a high specific capacity of 271.7 mAh g^−1^. The zinc-ion battery with the SEC as the cathode showed good mechanical properties, with a capacity retention rate of 71.9% under 300% tensile strain and 81.4% after 100 cycles at 200% strain.

#### Stretchable Supercapacitors

Energy storage in supercapacitors involves the absorption/desorption of ions or/and fast redox reactions at the electrode surface [379–385]. Like in stretchable batteries, SECs have also been mainly employed as the current collectors and electrodes in stretchable supercapacitors. (1) When serving as the current collectors, SECs are preferred to exhibit high conductivity and strong adhesion to the active electrodes [386–389]. Cui et al. [389] prepared an SEC by coating an LM onto a textile substrate and integrated it as the current collector, with CNTs as the active material and an ionic liquid polymer gel as the electrolyte to form a stretchable supercapacitor. The authors explored different textile substrates and found that EGain formed the most uniform and stable coating on polyester-based textiles. At 50% elongation, the resistance of the polyester-based SEC changed by less than 10% after 100 stretching/releasing cycles. The supercapacitor retained 92% of its initial capacitance at 200% strain, which can be equipped into clothing to provide a reliable and continuous power supply for LEDs during human movement. (2) When serving as the electrodes, SECs are favored to possess a large specific surface area, abundant electrochemically active sites, high conductivity, and adequate stretchability [263]. Wang et al. [263] deposited thiophene and 3-methyl thiophene on a stainless steel wire via electrochemical polymerization to prepare an SEC. The SEC exhibited a maximum tensile rate of 250% and was applied as an electrode in a stretchable supercapacitor, with a PVA/H_2_SO_4_ hydrogel as the electrolyte and PDMS as an encapsulation layer. The constructed supercapacitor maintained 93% of its capacitance after 10,000 stretching cycles to 100% strain.

The structural designs of SECs in stretchable supercapacitors, such as wavy, spiral, and core–shell structures, can help improve the devices’ tensile properties [390–392]. Lin et al. [392] developed an ant-nest amphiphilic polyurethane (sAPU) hydro-/organo-gel electrolyte, which interacted with carbon-based SECs as the electrodes for integrated stretchable supercapacitors (ISSCs) (Fig. 12f, g). A stretchable carbon-based electrode coated with sAPU fiber was prepared by electrospinning, and a porous interfacial layer was formed through layer folding and chemical crosslinking. This hybrid electrolyte–electrode structure was then immersed in a NaClO_4_/H_2_O/trimethyl phosphate electrolyte to create a flame-retardant integrated device. The 3D porous interface of this ant-like nest structure enhanced the toughness of the supercapacitor through mechanical meshing of the fiber and chemical crosslinking of the surface. The fabricated ISSC achieved a wide electrochemical window of 2.2 V, provided a high energy density of 13.7 mWh cm^−3^, and maintained 98.3% of its capacitance after 500 stretching/releasing cycles at 100% strain.

### Stretchable Sensors

In the realm of stretchable sensors and sensing systems, SECs have been commonly utilized as the sensing components or the electrodes, which play a vital role in realizing the basic functions and multi-functional integration of sensors for applications such as motion monitoring [248, 393–396], tactile sensing [397–399], and physiological signal monitoring [400–405].

SECs whose resistances change linearly with strain within a certain range have been applied as strain sensors in myriad areas [406] For example, Zhang et al. [406] utilized a self-healing maleic acid-grafted natural rubber/PANI/phytic acid (MNR/PANI/PA) SEC as a stretchable strain sensor. The device was prepared using a solution-processable method. Initially, maleic acid was grafted onto natural latex to form MNR. Aniline was then oxidized and polymerized in situ on the MNR template, with PA serving as both a crosslinking agent and a dopant. Finally, a uniform conductive film was formed by solution casting. The obtained SEC strain sensor exhibited high linearity (GF = 13.8@0–250% strain, GF = 32.0@250–1,000% strain), 1,000% stretchability, 2.5 MPa strength, and room-temperature self-healing capability.

**Fig. 13:**
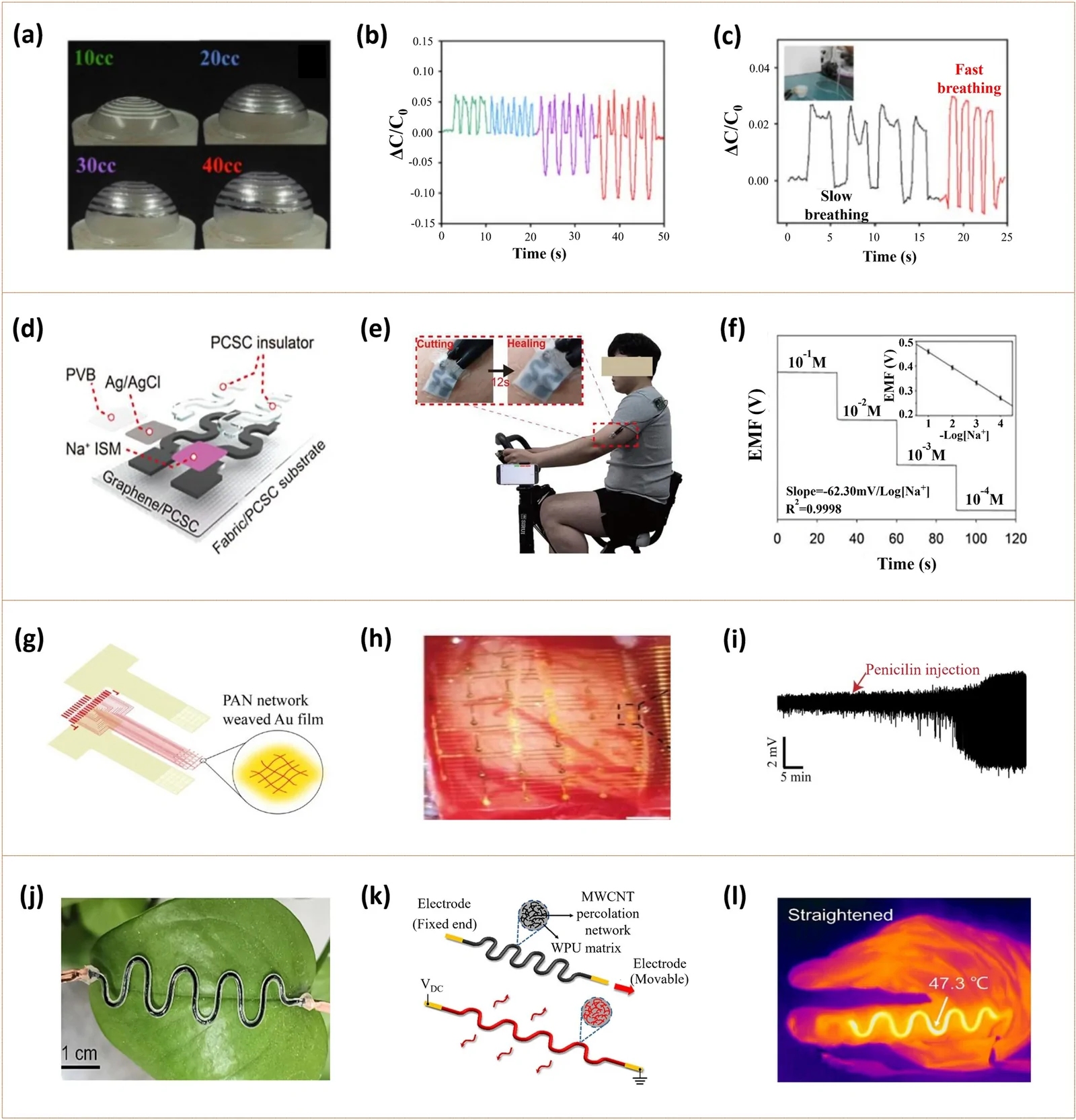
Applications in stretchable sensors and other applications. **a** A stretchable capacitive motion sensor with the LM/ExSil 100 SEC as the electrode expands under various volumes of inlet air. **b** Relative capacitance changes of the motion sensor with the SEC as the electrode during the injection and release of various air volumes. **c** The motion sensor with the SEC as the electrode detects respiration under various breathing modes. Reproduced with permission [411]. Copyright 2022, Elsevier B.V. **d** Schematic illustration of the snake-like P-Gr SEC-based electrochemical Na sensor. **e** Photograph of a person wearing a P-Gr SEC-based sensor during on-body and cutting/healing tests. **f** The electromotive force responses and a calibration curve of the P-Gr SEC-based sensor. Reproduced with permission [422]. Copyright 2022, Elsevier B.V. **g** Schematic illustration of the implantable stretchable sensor with PAN/Au SEC as the electrode. **h** PAN/Au SEC array conforms to the surface of the rat brain. Scale bar, 600 μm. **i** Real-time recording of the local field potential waveforms of the PAN/Au SEC. Reproduced with permission [425]. Copyright 2023, Korean Society of Medical and Biological Engineering. **j** Photograph of the serpentine SEC placed on a leaf. **k** Schematic illustration of the serpentine SEC composed of MWCNT percolation network on the WPU matrix as a stretchable heater. **l** IR image of the serpentine SEC heater at straightening. Reproduced with permission [439]. Copyright 2023, Elsevier B.V

For motion detection, stretchable sensors monitor and record various motion states in real time, which often involves mechanical deformation or abrasion. Applied in motion detection sensors, SECs are expected to exhibit stability under cyclic mechanical deformation and high wear resistance [407–411]. Tian et al. [410] developed an SEC based on a multistage composite structure as the core sensing element of a motion sensor. A three-step integrated process was employed to construct the gradient conductive network. (1) Flexible substrate construction: a TPU fabric with a bionic fiber interlace structure was prepared by electrospinning. (2) Conductive network optimization: AgNPs were loaded on the fiber surface via a dopamine-mediated in situ growth method, and acid-treated CNTs (ACNTs) were sprayed on the surface after plasma treatment to form an “AgNPs-ACNTs” bridging conductive pathway. (3) Interface function enhancement: fluorine CNTs/silica hybrid particles (FCNT-SIO_2_) were sprayed to construct a super-bisophobic surface with a concave corner structure. This multi-layer design enabled the SEC to achieve a tensile strength of 21.7 MPa and elongation at a break of 939% while maintaining a conductivity of 20.8 S cm^−1^. The motion sensor based on this SEC showed a wide detection range of 155% and a fast response time of 62 ms, maintaining stable signal output in extreme temperatures from − 60 to 60 °C and corrosive liquid environments, and successfully realizing real-time graded monitoring of a rider’s movement speed (slow, medium, and fast). Bhuyan et al. [411] fabricated an SEC composed of an uncrosslinked polysiloxane elastomer (ExSil 100) and a rheologically modified LM. The oxidized LM was coated on the surface of the elastomer layer using a template wetting method to obtain the SEC (Fig. 13a–c). The SEC served as the electrode of a capacitive motion sensor, and its excellent stretchability contributed to the sensor’s ability to detect the human body’s respiratory activity through the device’s volume change.

For tactile sensing, stretchable sensors respond to external stimuli and provide information such as pressure, temperature, humidity, and tangential strain, which can be employed in applications such as electronic skin and virtual reality [412–417]. They are often required to be capable of detecting weak stimuli signals. In this regard, SECs as the electrodes of tactile sensors are favored to have strain- or temperature-insensitive electrical properties, with their change in conductivity remaining within a certain range that does not affect the sensors’ sensing capability and adapting to a variety of external stimuli [418–420]. Kim et al. [420] introduced an SEC based on plasticized polyvinyl chloride (PVC) and graphene. The SEC was obtained by coating graphene on a glass substrate, spin-coating PVC gel on it, molding, and evaporating. The SEC could work normally under 50% tension. A TENG with this SEC as the electrode exhibited good biocompatibility and could act as a tactile sensor for the detection of the contact shape of objects.

For chemical detection, stretchable chemical sensors often detect chemicals in human fluids like sweat, and SECs applied in these sensors should possess high chemical stability and resistance to swelling in the presence of fluid or humidity [421, 422]. Son et al. [422] fabricated an SEC based on self-healing elastomers and graphene (Fig. 13d–f). In the first stage, a highly self-healing, conductive, and printable poly (1,4-cyclohexanedimethanol succinate-co-citrate)/graphene (P-Gr) ink was prepared through fluid-induced shearing and mixing. A snake-like SEC was then obtained by screen printing. It exhibited an electrical conductivity of 1,243 S m^−1^, self-healing properties (negligible resistance change in 10 cutting-healing cycles), and a stretchability of 213% tensile strain. The SEC was applied as the electrode of an ion sensor to detect Na^+^ in sweat, demonstrating good stability and high sensitivity (− 62.30 mV/log [Na^+^]).

For implantable stretchable sensors, they are implanted in vivo for detections such as the neural signal and epicardial signal [423, 424], and the employed SECs should exhibit excellent biocompatibility. Specifically, SECs for transient electronics should be capable of degrading in physiological environments in a controlled manner [425]. Yang et al. [425] manufactured an SEC by first preparing a polyacrylonitrile (PAN) nanofiber network through electrospinning and then depositing Au film via thermal evaporation (Fig. 13g–i). This SEC demonstrated high flexibility and low electrochemical impedance. This is because the PAN nanofiber network effectively prevents the Au film from cracking under strain and increases the surface roughness and effective active area of the Au film, which greatly reduces its impedance. The SEC was employed as the core recording electrode component of a microelectrode array, which was placed at the junction of a rat’s somatosensory cortex and motor cortex to record the increase in the amplitude and frequency of the neural signal.

### Other Applications

SECs have also found utility in other applications such as wearable heaters [426–431], antennas [432, 433], actuators for soft robotics or artificial muscles [434–436], and electromagnetic interference shielding [29, 66, 437, 438].

When applied as heating elements in wearable heaters, SECs efficiently convert electrical energy into thermal energy for local heating. This application necessitates good mechanical durability and thermal stability [439]. For example, Yuan et al. [439] developed a snake-like SEC composed of MWCNTs/WPU nanocomposite yarns and applied it as a wearable heating device (Fig. 13j–l). The preparation process of the SEC-based wearable heater involved two steps. (1) The nanocomposite conductive yarn (MWCNTs/WPU) was prepared via a wet spinning process, followed by a stress drying method to promote the densification of the conductive network. (2) The conductive yarn was encapsulated in a thick WPU elastic sheath through solution impregnation combined with thermal curing, and then, the serpentine structure was formed using 3D printing templates. This strain-insensitive SEC-based wearable heater exhibited high electrical stability (Δ*R*/*R*_0_ < 1.6% at 100% strain) while achieving rapid joule heating to 47 °C in 90 s at 15 V, with waterproof property and self-healing capability.

When applied as radiation elements in an antenna, SECs are capable of transmitting or receiving electromagnetic wave signals to support wireless communication [432, 433, 440]. This application demands SECs with high conductivity and resistance to environmental interference. A key challenge is the declined wireless performance under strain, which can be alleviated through strategies like exploiting a “dielectro-elastic” composite substrate with tunable dielectric properties to offset resonance frequency shifts [441]. He et al. [440] developed a biomimetic SEC based on a spider web architecture and LM microchannel as the core component of a near-field communication antenna. The SEC was prepared via a 3D direct-write printing process. To begin with, EGaIn was uniformly dispersed in a silicone elastomeric matrix to form a printable composite ink. A spider-shaped serpentine web was then constructed via 3D printing. Subsequently, an ultra-thin LM conductive channel encapsulated by a protective silicone barrier was prepared using a peeling-off activation strategy. The architecture achieved high electromechanical stability, maintaining reliable energy transmission and information communication with a minimum resonant frequency shift of 2.75 MHz at 300% tensile strain, as well as strong performance through 5,000 stretching/releasing cycles at 100% strain and complex deformations including 170° folding, 270° twisting, and 360° rolling.

When applied as actuators for soft robotics or artificial muscles, based on different actuating mechanisms, the SECs may serve as the electrodes or functional materials (e.g., as the heater for heat-driven actuators), high conductivity or high mechanical and thermal stability are required for such application scenarios. When applied for electromagnetic interference shielding, high conductivity is the main key for the SECs to achieve high shielding, and nanostructure designs to reduce the reflection is conducive to high efficiency of electromagnetic interference shielding.

## Summary of This Chapter

The applications of SECs cover the three core areas of energy conversion, energy storage, and sensing. (1) In applications of energy conversion, SECs can be applied for nanogenerators, solar cells, and fuel cells. SECs for PENGs demand high cyclic strain tolerance and mechanical durability; SECs as the triboelectric layer of TENGs require high wear resistance; and SECs for pyroelectric and thermoelectric nanogenerators necessitate high thermal stability. In solar cells, SECs can serve as the transparent electrodes (require high transparency and high conductivity), back electrodes (require high conductivity and prefer high reflection of light), interconnecting components (require high conductivity and mechanical strength under tension), or photoactive layers (require high light absorption efficiency, mechanical stability, and chemical compatibility). In fuel cells, SECs can serve as the electrodes, current collectors, and interconnecting components. As the electrodes, they need to be electrochemically active and sufficiently conductive. As the current collectors, high conductivity and mechanical adaptability are essential. As interconnecting components, strain-insensitive conductivity and fatigue resistance are favored. (2) In applications of energy storage, SECs can be applied as the electrodes and current collectors for batteries and supercapacitors. When employed as the current collectors, high conductivity and good interface bonding force are preferred. When employed as the electrodes, large specific surface area, good conductivity, and high electrochemical activity are favored. (3) In applications of sensing, SECs can be applied for various kinds of sensors to detect motion, tactile, chemical, etc., serving as the sensing components or electrodes. In motion detection sensors, SECs are required to have high mechanical stability and wear resistance under cyclic mechanical deformation. In tactile sensors, SECs are required to have high electrical stability and stimulus adaptability. In chemical detection sensors, SECs are required to possess high chemical stability and anti-swelling property. In implantable sensors, SECs must possess high biocompatibility. (4) In addition, SECs have other applications such as wearable heating devices antennas, actuators, and electromagnetic interference shielding. In wearable heating devices, SECs need to have good mechanical durability and thermal stability. In antennas, SECs need to possess high electrical conductivity and resistance to environmental interference. In actuators for soft robotics or artificial muscles, high conductivity or high mechanical and thermal stability are needed based on different actuating mechanisms. For electromagnetic interference shielding, high electrical conductivity is required and nanostructure designs to reduce reflection are favorable. Table 4 summarizes the diverse applications of SECs.

**Table 4:** Diverse applications of SECs

Application	Device type	Function of the SECs	Requirement
Stretchable energy conversion devices	Triboelectric nanogenerator	Electrode	High mechanical strength, fair conductivity and high stability
		Triboelectric layer	Strong abrasion resistance, high mechanical stability
	Piezoelectric nanogenerator	Electrode/piezoelectric material	High mechanical strength, high cyclic durability under strain
	Pyroelectric nanogenerator	Electrode/pyroelectric material	High thermal stability and durability
	Thermoelectric nanogenerator	Electrode	High thermal stability, high conductivity and stability
		Thermoelectric material	High Seebeck coefficient, high electrical conductivity, and low thermal conductivity
	Solar cell	Transparent electrode	High transparency and electrical conductivity
		Back electrode	High conductivity and high reflectivity
		Interconnecting component	Sufficient stretchability, high mechanical strength
		Photoactive layer	High optical absorption and charge transport capabilities, high mechanical stability, and high chemical durability
	Fuel cell	Electrode	High conductivity, corrosion resistance
		Current collector	High conductivity, low contact resistance, high mechanical durability
		Interconnecting component	Gas impermeability, thermal stability, low interfacial resistance
Stretchable energy storage devices	Battery	Electrode	Good conductivity, high electrochemical stability, and good tensile properties
		Current collector	Ultra-low resistance, strong adhesion to active electrodes, good stretchability
	Supercapacitor	Electrode	High specific surface area and fast charge transfer process
		Current collector	High conductivity, strong adhesion to active electrodes, and good stretchability
Stretchable Sensors	Motion monitoring sensor	Electrode/sensing component	High stability and high wear resistance
	Tactile sensor	Electrode/sensing component	Strain- or temperature- insensitive electrical properties and high conductivity as the electrode; high sensitivity and stability as the sensing component
	Chemical sensor	Electrode/sensing component	High conductivity and chemical stability
	Implantable stretchable sensor	Electrode/sensing component	Excellent biocompatibility, high conductivity and good stretchability
	Strain sensor	Sensing component	High sensitivity and strain-resistance linear response, high durability under cyclic strain
		Electrode	High conductivity, strain-insensitive property
Other applications	Wearable heater	Heating element	High mechanical durability and thermal stability
	Communication antenna	Radiation element	High conductivity and anti-environmental interference ability
	Actuator	Electrode	Sufficient stretchability and high conductivity
		Functional component	Sufficient stretchability; high mechanical and thermal stability (heat driven)
	Electromagnetic interference shielding	Functional materials	High conductivity and sufficient stretchability

## Conclusion and Future Prospects

As an indispensable base material of stretchable electronics, SECs have become a research hotspot in recent years. SECs can take into account both mechanical stretchability and electrical properties, enabling electronic devices to adapt to complex application scenarios involving various deformations. This capability is poised to promote the practical implementation of stretchable electronics in myriad areas including medical care, robotics, sports, and entertainment.

SECs can be divided into metal-based, inorganic nonmetallic materials-based, conductive polymer-based, and composite materials-based SECs based on their primary conductive components. (1) Metal-based SECs. Solid metal materials are generally processed into various nanostructures, subsequently manufactured into designed shapes, and combined with stretchable substrates to create SECs. LM generally requires a combination with a supporting polymer matrix to form an SEC, with preparation methods including microchannel injection, adhesion and patterning on the surface of an elastomer, and self-assembly of modified LMs into films. (2) Inorganic nonmetallic materials-based SECs. Inorganic nonmetallic nanomaterials can achieve stretchability through integration with elastomers, or combination with high-aspect-ratio nanomaterials to form multilayer or network architectures, which can be prepared through techniques such as solution spin coating, vacuum filtration, layer-by-layer self-assembly and decal transfer. (3) Conductive polymer-based SECs. PEDOT:PSS is the predominantly employed conductive polymer, whose tensile properties can be improved by incorporating small molecule plasticizers or surfactants, and conductivity can be enhanced by doping with substances like polar solvents, strong acids, and ionic liquids. Conductive polymer-based SECs can be mainly prepared by solution treatment, in-situ polymerization and laser-induced techniques. (4) Composite materials-based SECs. They can achieve enhanced conductivity and stretchability by means of multi-packing coordination, nano-size regulation, and double-ligand surface modification, whose most common preparation technique is solution mixing and molding, with uniform dispersion of conductive fillers being a critical factor. In addition, electrostatic spinning, screen printing, and 3D printing can be employed to create SECs with diverse structures. The stretchability of SECs can also be achieved through structural design strategies, the core principle of which is to maintain the continuity of the conductive pathways by dispersing the external stress through geometric deformation. The applications of SECs span the three core areas of energy conversion, energy storage, and sensing and extend to stretchable heaters, antennas and electromagnetic interference shielding. In energy conversion devices, they primarily function as the electrodes or functional active layers. In energy storage devices, they can serve as the current collectors or active electrodes. In sensing applications, they can act as the sensing elements or electrical signal transmission media.

At present, SECs still face tough challenges, such as conductivity loss upon applied strain, susceptibility to external environmental factors, undesirable performance stability, and high cost. (1) The electronic structures within the SEC are often altered or damaged during deformation, resulting in conductivity loss under strain. (2) Surrounding environmental stimuli such as temperature, pressure and humidity could also impact the conductivity of SECs, while prolonged exposure to ultraviolet light or atmospheric environment could induce degradation or oxidation, impairing the SEC’s electrical and mechanical stability. (3) The fatigue-induced performance decrements under repeated stretching/releasing cycles and the performance decline due to long-term gradual oxidation or degradation are also critical concerns. (4) Concerning the cost, the prices of raw materials like liquid metal, Ag NWs, CNTs, MXenes, and PEDOT:PSS for the preparation of SECs are relatively high, and the production costs would be elevated to a level out of ordinary consumers’ reach if superimposing expensive fabrication processes, which limit their large-scale production. To address these challenges, it is necessary to explore novel materials, preparation processes, and design routes. (1) As for overcoming the problem of conductivity under strain, the key is to maintain a connected conductive path within the SEC under strain, which could be achieved through structural design of conductive components to counteract stress with geometric deformation or material design to separate the continuous conductive phase with the supporting stretchable polymer phase. (2) As for the surrounding environmental effects, a combination of conductive components with positive and negative temperature coefficients could circumvent the problem of the intrinsic conductivity variation of a single conductive component to temperature change, and a polymer substrate or matrix prepared by binary or multiple polymer components with positive and negative thermal expansion coefficients could be a solution for the conductivity change caused by the volume change of the polymer substrate or matrix with changing temperature. Material protection of a polymer with a higher Young’s modulus for the SEC could avoid the impact of pressure, and surface modification with a superhydrophobic effect or outside packaging layer for the SEC could circumvent the influence of humidity or atmosphere. (3) As for the undesirable performance stability, material design with adjustment of the micro-/nano-structure and composition could be explored to improve the fatigue performance and long-term stability. (4) As for the high cost, developing efficient fabrication techniques and refining process parameters could increase the production efficiency, reduce the fabrication costs, and promote the large-scale production. With continued research progress and industrial development in these areas, both the performance and application potential of SECs will keep enhancing and expanding.

In summary, the rapid development of stretchable and wearable electronics pushes up the refinement and improvement in SECs while the momentous advancement of SECs fuels the birth of new-generation electronics and technologies. The SECs hold bright prospects and a prosperous future, with great application potential and huge market value. The realization of such visions calls for the collaborative efforts and support of researchers, enterprises, and governments.

## Abbreviations

SECs: Stretchable electronic conductors
Cu: Copper
Au: Gold
Ag: Silver
PDMS: Polydimethylsiloxane
NWs: Nanowires
EGaIn: Eutectic gallium indium
SHL-LIG: Super-hydrophilic laser-induced graphene
PI: Polyimide
H: Hydrogen
LM: Liquid metal
InOG: Indium/oxide film/gallium
LMPs: Liquid metal particles
In_2_O_3_: Indium oxide
1D: One-dimensional
2D: Two-dimensional
3D: Three-dimensional
CNTs: Carbon nanotubes
BBE: Bottle brush elastomer
SWCNTs: Single-walled carbon nanotubes
CNF: Carbon nanofiber
PAAm: Polyacrylamide
AAm: Acrylamide
PANI: Polyaniline
PVA: Polyvinyl alcohol
PL-MXene: Polymer-laminated-MXene
PEDOT:PSS: Poly (3,4-vinyldioxythiophene): poly (styrene sulfonic acid)
PEO: Polyethylene oxide
DMSO: Dimethyl sulfoxide
PES: Polyether sulfonate
GEM: General effective medium
WPU: Waterborne polyurethane
TPU: Thermoplastic polyurethane
In: Indium
Ga: Gallium
STNNE: Stretchable transparent nanofiber network electrode
rGO: Reduced graphene oxide
AgNPs: Silver nanoparticles
SEBS: Poly(styrene ethylene butylene styrene)
BC: Bacterial cellulose
Ni: Nickel
CVD: Chemical vapor deposition
LM-Eke: Liquid metal-coated elastic kirigami electrode
MM: Mechanical metamaterial
MWCNTs: Multiwalled carbon nanotubes
DEG: Diethylene glycol
CHCl_3_: Chloroform
ELD: Electroless deposition
PVP: Polyvinylpyrrolidone
MGG: Multi-layer graphene/graphene vortex
PMMA: Polymethyl methacrylate
PENG: Piezoelectric nanogenerator
TENG: Triboelectric nanogenerator
PN: Percolation network
SIBS: Poly(styrene-isobutylene-styrene)
PC: Polycarbonate
APBC: (4-Aminotetrahydropyran)_2_PbBr_2_Cl_2_
P(VDF-TrFE): Poly(vinylidene fluoride-trifluoroethylene)
SOSCs: Stretchable organic solar cells
D18: Poly[(2,6-(4,8-bis(5-(2-ethylhexyl-3-fluoro)thiophen-2-yl)-benzo[1,2-b:4,5-b’]dithiophene))-alt-5,5’-(5,8-bis(4-(2-butyloctyl)thiophen-2-yl)dithieno[3’,2’:3,4;2”,3”:5,6]benzo [1,2-c][1,2,5]thiadiazole)]
PEHDT: Poly[bis(2-hexyldecyl) 5-(4,8-bis(5-(2-ethylhexyl)-4-fluorothiophen-2-yl)-6-methylbenzo[1,2-b:4,5-b′]dithiophen-2-yl)-5″-methyl-[2,2′:5′,2″-terthiophene]-3,3″-dicarboxylate]
v-AuNWs: Vertically aligned gold nanowires
CY: Carbon yarn
sAPU: Ant-nest amphiphilic polyurethane
ISSC: Integrated stretchable supercapacitor
ACNTs: Acid-treated carbon nanotubes
FCNTs: Fluorinated carbon nanotubes
PVC: Polyvinyl chloride
PAN: Polyacrylonitrile
